# Novel Organic Salts Based on Mefloquine: Synthesis, Solubility, Permeability, and In Vitro Activity against *Mycobacterium tuberculosis*

**DOI:** 10.3390/molecules27165167

**Published:** 2022-08-13

**Authors:** Dário Silva, Márcio V. C. Lopes, Željko Petrovski, Miguel M. Santos, Jussevania P. Santos, Sueli F. Yamada-Ogatta, Marcelle L. F. Bispo, Marcus V. N. de Souza, Ana Rita C. Duarte, Maria C. S. Lourenço, Raoni Schroeder B. Gonçalves, Luis C. Branco

**Affiliations:** 1 LAQV-REQUIMTE, Department of Chemistry, NOVA School of Science and Technology, 2829-516 Caparica, Portugal; 2 Instituto de Química, Universidade Federal do Rio de Janeiro, Av. Athos da Silveira Ramos 149, Cidade Universitaria, Rio de Janeiro 21941-909, Brazil; 3 Departamento de Microbiologia, Universidade Estadual de Londrina, Rodovia Celso Garcia Cid (PR 445), Km 380, Campus Universitário, Londrina 86057-970, Brazil; 4 FioCruz-Fundação Oswaldo Cruz, Instituto de Tecnologia em Fármacos-Far-Manguinhos, Rua Sizenando Nabuco, 100, Manguinhos, Rio de Janeiro 21041-250, Brazil; 5 Instituto de Pesquisas Clínica Evandro Chagas—IPEC, Av. Brasil, 4365, Manguinhos, Rio de Janeiro 21040-900, Brazil

**Keywords:** API-OSILs, ionic liquids, mefloquine, polymorphism, tuberculosis

## Abstract

The development of novel pharmaceutical tools to efficiently tackle tuberculosis is the order of the day due to the rapid development of resistant strains of *Mycobacterium tuberculosis*. Herein, we report novel potential formulations of a repurposed drug, the antimalarial mefloquine (MFL), which was combined with organic anions as chemical adjuvants. Eight mefloquine organic salts were obtained by ion metathesis reaction between mefloquine hydrochloride ([MFLH][Cl]) and several organic acid sodium salts in high yields. One of the salts, mefloquine mesylate ([MFLH][MsO]), presented increased water solubility in comparison with [MFLH][Cl]. Moreover, all salts with the exception of mefloquine docusate ([MFLH][AOT]) showed improved permeability and diffusion through synthetic membranes. Finally, in vitro activity studies against *Mycobacterium tuberculosis* revealed that these ionic formulations exhibited up to 1.5-times lower MIC values when compared with [MFLH][Cl], particularly mefloquine camphorsulfonates ([MFLH][(1*R*)-CSA], [MFLH][(1*S*)-CSA]) and mefloquine HEPES ([MFLH][HEPES]).

## 1. Introduction

Tuberculosis (TB) remains a major public health concern and currently is the leading cause of human death by an infectious disease. According to the World Health Organization, around 10 million people were infected with the *Mycobacterium tuberculosis* in 2018 and 1.5 million deceased [1]. The emergence and spread of multidrug-resistant (MDR) and extensively drug-resistant (XDR) strains is particularly alarming. The conventional treatment for MDR-TB may take up to 24 months and only 56% of the cases are successfully treated [2,3,4]. Therefore, the development of new TB agents that result in shorter and more effective treatments is an urgent need.

Drug repurposing has emerged as an important tool in the fight against TB [5,6,7]. This strategy is characterized by identifying new applications for approved or under-investigation drugs that are different from their initial scope [8,9,10,11]. The main advantage is that the pharmacological properties of the substance, such as tissue distribution, maximum serum concentration, and metabolism pathways, are already described, and the drug’s safety profile was properly established. It makes the development process faster and less costly, which can be particularly useful for the discovery of antibiotics, due to the fast development of resistance and the low interest in investments as a result of the usually nonattractive return [12].

In the current pipeline for the development of new anti-TB treatments, six of the twenty-one substances are repurposed drugs (auranofin, nitazoxanide, linezolid, clofazimine, moxifloxacin, and levofloxacin). Among them, four are in phase 3 clinical trials [13,14,15,16]. In addition, several other substances primarily developed for other diseases have been investigated in vitro and in vivo against TB in pre-clinical assays [17].

Mefloquine (MFL), a widely used drug for malaria treatment and prophylaxis [18,19], has presented very interesting results against TB. This substance was originally developed as an antimalarial agent by the US army in the 1970s, during the Vietnam War, and was registered by the United States Food and Drug Administration in 1989 [20]. MFL presents a broad-range activity against Gram-positive bacteria, such as *Streptococcus*, *Staphylococcus*, and *Pneumococcus* spp., including methicillin-resistant strains, and it is also active against Gram-negative bacteria such as *Escherichia coli* and *Klebsiella pneumoniae* [21,22,23,24]. The first reports against *Mycobacterium* species were presented by Bermurdes and coworkers, where the authors demonstrated the in vitro and in vivo activity against susceptible and macrolide-resistant strains of *M. avium* [25,26]. With regard to *M. tuberculosis*, Jayaprakash and coworkers reported that MFL has a relatively high activity against the nonreplicating persistent phenotype and, as observed against malaria, the *erythro* isomers were more active against *M. tuberculosis* than the *threo* isomers [27]. Bermudez and Meek displayed the bactericidal activity of MFL against *M. tuberculosis* in acid and low-oxygen media, to simulate the environments found in the phagocyte vacuole and in granulomas. The authors also demonstrated that MFL is significantly active against intracellular *M. tuberculosis* H37Rv in a macrophage infection model and is not cytotoxic to the host cell [28]. In a previous work published by our group, we demonstrated that MFL is active against the MDR-TB strain T113 (which is resistant to isoniazid, rifampicin, ethambutol, and ofloxacin) and the MIC was identical to the one observed for the susceptible strain H37Rv [29]. Furthermore, it was active in vivo, in a murine model of TB after administration by the oral route and displayed an in vitro synergistic activity with isoniazid [30]. Thus, the use of MFL for the development of new treatment regimens against TB and the synthesis of more potent derivatives is a promising research area [31,32,33,34,35].

During recent years, the use of organic salts and ionic liquids (OSILs) has emerged as a topic of intense scientific interest. Due to the unique characteristics of these substances, such as negligible vapor pressure, good thermal stability, high electric conductivity, and miscibility with water and organic solvents, applications in different fields have been described. Initially, Ionic Liquids (ILs), as organic salts with low melting temperatures (m.p. < 100 °C), were widely explored as green solvents for chemical reactions, particularly room-temperature ionic liquids (RTILs). Then, functional OSILs were designed for applications in materials science, catalysis, electrochemistry, biotechnology, analytics, among others [36,37,38,39,40]. However, it was just in recent years that the development of OSILs containing bioactive cations or anions began to attract attention and the third generation of ILs emerged with important applications in the pharmaceutical area [41,42,43,44,45,46,47,48,49,50].

The conversion of a bioactive compound or an active pharmaceutical ingredient (API) into an API-OSIL allows the fine-tuning of their physicochemical and pharmacological properties, leading to the development of substances with improved water solubility, membrane permeability, bioavailability, and biological activity. Particularly, with regard to the development of antibiotics, our group has recently studied the properties of fluoroquinolone (ciprofloxacin and norfloxacin) [51,52,53] and β-lactam (penicillin G and amoxicillin hydrolysates, and ampicillin) [54,55] API-OSILs and their biological activities against susceptible and resistant bacteria strains. We demonstrated that properties such as water solubility and the octanol-water partition coefficient could be significantly modulated according to the choice of counter-ions. Furthermore, the synergic interaction between the ionic species led to an improvement in the antimicrobial activities, particularly of the β-lactam-based OSILs. These salts showed activity against resistant strains of *Escherichia coli* and *Staphylococcus aureus* in comparison with the parent APIs, with minimum inhibitory concentration values similar to the ones found against the susceptible strains.

Hence, considering the potential of MFL for the development of new treatments against TB and the emergence of API-OSILs as an important strategy to achieve improved pharmaceutical properties of drugs, herein, we present our work on organic salts based on MFL as an improved formulation of this drug to tackle *M. tuberculosis*.

## 2. Materials and Methods

Commercially available reagents were purchased from Sigma-Aldrich, Alfa Aesar, and TCI. Solvents were purchased from LabChem. ^1^H and ^13^C NMR spectra in (CD_3_)_2_SO (from Euriso-Top) were recorded on a Bruker AMX 400 spectrometer at room temperature unless specified otherwise. To perform NMR, 5 mm borosilicate tubes were used and the sample concentration was, approximately, 7 mg/mL to ^1^H NMR and 30 mg/mL to ^13^C NMR. Chemical shifts are reported upfield in parts per million (ppm) in reference to the residual nondeuterated solvent. The elemental analysis experiments were performed in a Thermo Finnigan-CE Instruments Flash EA 1112 CHNS series under standard conditions (T combustion reactor 900 °C, T GC column furnace 65 °C, multiseparation SS GC column, He flow 130 mL/min, and O_2_ flow 250 mL/min) at the REQUIMTE Analysis Lab, Departamento de Química Faculdade de Ciências e Tecnologia. FTIR spectra of the samples were recorded on a Perkin-Elmer FT-IR Spectrometer Spectrum Two (Waltham, MA, USA), equipped with an attenuated total reflection (ATR) cell in the range of 4000–400 cm^−1^. DSC thermal studies were carried out using a TA Instruments Q-series TM Q2000 DSC with a refrigerated cooling system. The sample was continuously purged with a 50 mL/min nitrogen flow. About 5 to 10 mg of each FQ-OSIL was crimped in an aluminum standard sample pan with a pinhole lid.

### 2.1. Methods

All MFL organic salts were prepared according to the following general procedure: to a magnetically stirred solution of mefloquine hydrochloride in ethanol (50 mg/mL), the organic acid sodium salt (1.0 equivalent with regard to mefloquine hydrochloride) was added dissolved in ethanol. After 16 h at room temperature, the reaction mixture was filtered to remove the formed sodium chloride and the solvent was subsequently removed under reduced pressure to obtain the desired product.

2-((2,8-*bi*s(trifluoromethyl)quinolin-4-yl)(hydroxy)methyl)piperidin-1-ium 1,4-*bis*(2-ethylhexoxy)- -1,4-dioxobutane-2-sulfonate, **[MFLH][AOT]**

Following the general procedure, sodium docusate (1.05 g; 2.36 mmol) was added to a solution of mefloquine hydrochloride. White solid (92%). T_m_ = 125.1 °C.

^1^H NMR (400.13 MHz, DMSO-*d_6_*) δ(ppm): 8.88 (d, J = 8.8 Hz, 1H); 8.40 (d, J = 7.2 Hz, 1H); 8.09 (s, 1H); 7.99 (t, J = 16 Hz, 1H); 6.00 (s, 1H); 3.92-3.84 (m, 4H); 3.63 (dd, J = 11,6 Hz, J = 3,6 Hz, 1H); 3.25 (d, J = 12.4 Hz, 1H); 2.99-2.76 (m, 3H); 1.69-1.46 (m, 7H); 1.35-1.22 (m, 18H); 0.86-0.80 (m, 12H). ^13^C NMR (100.63 MHz, DMSO-*d_6_*) δ(ppm): 171.09; 168.39; 151.15; 142.84; 129.16; 128.47; 126.46; 115.48; 67.64; 66.31; 66.24; 66.19; 61.51; 58.71; 44.28; 38.19; 34.15; 29.80; 29.68; 29.62; 28.40; 23.26; 23.05; 22.48; 22.45; 21.62; 21.20; 21.05; 13.98; 13.95; 10.88; 10.84; 10.80. FTIR-ATR υ (cm^−1^): 2961; 2933; 2872; 1728; 1589; 1456; 1344; 1306; 1244; 1183; 1156; 1139; 1106; 1039; 1005; 1917; 894; 833; 772; 739; 717; 667; 528. Elemental analysis calcd for C_37_H_54_F_6_N_2_O_8_S⋅3H_2_O: C 51.98; H 7.07; N 3.28. Found: C 51.4; H 6.37; N 3.03.

2-((2,8-*bis*(trifluoromethyl)quinolin-4-yl)(hydroxy)methyl)piperidin-1-ium methanesulfonate, **[MFLH][MsO]**

Following the general procedure, sodium mesylate (50.7 μL; 0.78 mmol) was added to a solution of mefloquine hydrochloride. White solid (71%). T_m_ = 173.2; 210.1 °C. ^1^H NMR (400.13 MHz, DMSO-*d_6_*) δ(ppm): 8.60 (d, J = 8.4 Hz, 1H); 8.39 (d, J = 7.2 Hz, 1H); 8.07 (s, 1H); 7.99 (t, J = 8 Hz, 1H); 5.79 (s, 1H); 3.48 (d, J = 11.6 Hz, 1H); 3.29 (d, J = 10.8 Hz, 1H); 2.98 (t, J = 10.8 Hz, 1H); 2.40 (s, 3H); 1.69-1.54 (m, 4H); 1.24-1.18 (m, 2H). ^13^C NMR (100.63 MHz, DMSO-d_6_) δ(ppm): 150.98; 147.41; 143.20; 130.51; 129.03; 128.94; 126.72; 115.97; 68.16; 59.20; 44.89; 21.87; 21.65; 21.53. FTIR-ATR υ (cm^−1^): 2964; 2863; 1313; 1206; 1192; 1134; 1109; 1043; 1011; 899; 833; 795; 775; 738; 718; 669; 643; 557; 537; 522; 445. Elemental analysis calcd for C_18_H_20_F_6_N_2_O_4_S: C 45.56; H 4.25; N 5.90. Found: C 45.67; H 4.67; N 5.47.

2-((2,8-*bis*(trifluoromethyl)quinolin-4-yl)(hydroxy)methyl)piperidin-1-ium (1*S*)-2-oxo-bornane- -10-sulfonate, **[MFLH][(1*S*)-CSA]**

Following the general procedure, sodium (1*S*)-camphorsulfonate (0.16 g; 0.69 mmol) was added to a solution of mefloquine hydrochloride. White solid (91%). ^1^H NMR (400.13 MHz, DMSO-*d_6_*) δ(ppm): 9.91 (s br, 1H); 8.90 (d, J = 8.4 Hz, 1H); 8.40 (d, J = 7.2 Hz, 1H); 8.09 (s, 1H); 7.98 (t, J = 7.6 Hz, 1H); 6.81 (d, J = 4 Hz, OH, 1H); 6.02 (s, 1H); 3.46-3.41 (m, 1H); 3.26 (d, J = 12.4 Hz, 2H); 2.98-2.95 (m, 1H); 2.89 (d, J = 14.8 Hz, 1H); 2.70-2.62 (m, 1H); 2.38 (d, J = 14.8 Hz, 1H); 2.23 (dt, J^1^ = 18 Hz, J^2^ = 3.6 Hz, 1H); 1.93 (t, J = 4.8 Hz, 1H); 1.89-1.77 (m, 2H); 1.65-1.58 (m, 4H); 1.32-1.23 (m, 4H); 1.04 (s, 3H); 0.74 (s, 3H). ^13^C NMR (100.63 MHz, DMSO-*d_6_*) δ(ppm): 216.31; 146.43; 142.75; 129.79; 128.03; 126.75; 119.89; 115.55; 69.30; 62.01; 59.93; 58.22; 47.06; 46.69; 45.22; 42.25; 42.13; 26.39; 25.48; 24.15; 23.25; 22.24; 20.09; 19.54. FTIR-ATR υ (cm^−1^): 2964; 2869; 1738; 1598; 1307; 1184; 1139; 1105; 1038; 932; 837; 798; 775; 737; 714; 667; 615; 600; 581; 527; 511. Elemental analysis calcd for C_27_H_32_F_6_N_2_O_4_S⋅2H_2_O: C 50.15; H 5.61; N 4.33. Found: C 50.29; H 5.7; N 4.12.

2-((2,8-*bis*(trifluoromethyl)quinolin-4-yl)(hydroxy)methyl)piperidin-1-ium (1*R*)-2-oxo-bornane-10-sulfonate, **[MFLH][(1*R*)-CSA]**

Following the general procedure, sodium (1*R*)-camphorsulfonate (0.16 g; 0.69 mmol) was added to a solution of mefloquine hydrochloride. White solid (98%). ^1^H NMR (400.13 MHz, DMSO-*d_6_*) δ(ppm): 9.81 (s br, 1H); 8.87 (d, J = 8.4 Hz, 1H); 8.40 (d, J = 7.2 Hz, 1H); 8.09 (s, 1H); 7.98 (t, J = 8.0 Hz, 1H); 6.81 (d, J = 4 Hz, OH, 1H); 6.0 (s, 1H); 3.45-3.42 (m, 1H); 3.26 (d, J = 12.4 Hz, 1H); 2.96 (t, J = 11.2 Hz, 1H); 2.88 (d, J = 14.8 Hz, 1H); 2.70-2.63 (m, 1H); 2.38 (d, J = 14.8 Hz, 1H); 2.23 (dt, J^1^ = 18.4 Hz, J^2^ = 4.0 Hz, 1H); 1.93 (t, J = 4.4 Hz, 1H); 1.85-1.76 (m, 2H); 1.65-1.57 (m, 4H); 1.31-1.22 (m, 4H); 1.03 (s, 3H); 0.73 (s, 3H). ^13^C NMR (100.63 MHz, DMSO-*d_6_*) δ(ppm): 216.30; 151.17; 145.38; 142.78; 129.12; 128.39; 126.41; 125.04; 119.69; 115.45; 67.69; 58.81; 58.21; 47.08; 46.72; 44.42; 42.25; 42.13; 26.39; 24.16; 21.57; 21.15; 20.98; 20.07; 19.53. FTIR-ATR υ (cm^−1^): 2958; 2869; 1738; 1601; 1585; 1432; 1309; 1142; 1107; 1037; 967; 931; 894; 865; 836; 772; 737; 714; 667; 613; 600; 581; 529; 512; 442. Elemental analysis calcd for C_27_H_32_F_6_N_2_O_4_S⋅1.5H_2_O: C 50.86; H 5.53; N 4.39. Found: C 50.76; H 5.06; N 4.38.

2-((2,8-*bis*(trifluoromethyl)quinolin-4-yl)(hydroxy)methyl)piperidin-1-ium 1,1-dioxo-1,2-benzo- -thiazol-3-olate, **[MFLH][Sac]**

Following the general procedure, sodium saccharinate (0.156 g; 0.75 mmol) was added to a solution of mefloquine hydrochloride. White solid (74%). T_m_ = 252.2 °C. ^1^H NMR (400.13 MHz, DMSO-*d_6_*) δ(ppm): 9.10 (s br, 1H); 8.67 (d, J = 8.8 Hz, 1H); 8.41 (d, J = 7.2 Hz, 1H); 8.11 (s, 1H); 8.01 (t, J = 8.0 Hz, 1H); 7.67-7.57 (m, 4H); 6.81 (d, J = 4.4 Hz, OH, 1H); 5.84 (s, 1H); 3.52 (d, J = 11.6 Hz, 1H); 3.03 (t, J = 12 Hz, 1H); 1.70-1.58 (m, 4H); 1.30-1.22 (m, 2H); 1.03 (d, J = 6.0 Hz, 1H). ^13^C NMR (100.63 MHz, DMSO-*d_6_*) δ(ppm): 167.97; 150.84; 146.53; 145.25; 142.76; 134.71; 131.58; 129.94; 128.69; 128.41; 127.08; 126.34; 122.50; 119.82; 119.14; 115.53; 67.88; 62.15; 58.94; 44.54; 25.48; 21.58; 21.23; 21.18. FTIR-ATR υ (cm^−1^): 2970; 2863; 1604; 1617; 1575; 1457; 1429; 1315; 1282; 1193; 1148; 1116; 1103; 1055; 1007; 951; 839; 794; 778; 753; 741; 729; 703; 680; 663; 650; 635; 602; 601; 543; 528; 440. Elemental analysis calcd for C_24_H_21_F_6_N_3_O_4_S: C 51.34; H 3.77; N 7.48. Found: C 51.11; H 4.0; N 7.21.

2-((2,8-*bis*(trifluoromethyl)quinolin-4-yl)(hydroxy)methyl)piperidin-1-ium 4-methylbenzene- -sulfonate, **[MFLH][TsO]**

Following the general procedure, sodium tosylate (0.196 g; 0.73 mmol) was added to a solution of mefloquine hydrochloride. White solid (75%). T_m_ = 250.6 °C. ^1^H NMR (400.13 MHz, DMSO-*d_6_*) δ(ppm): 8.81 (d, J = 8.8 Hz, 1H); 8.37 (d, J = 7.2 Hz, 1H); 8.07 (s, 1H); 7.87 (t, J = 8.0 Hz, 1H); 7.43 (d, J = 7.6 Hz, 2H); 7.13 (d, J = 8.0 Hz, 2H); 5.94 (s, 1H); 3.45-3.33 (m, 4H); 3.15 (d, J = 12.4 Hz, 1H); 2.84 (t, J = 11.6 Hz, 1H); 2.27 (s, 3H); 1.63-1.54 (m, 4H); 1.23-1.00 (m, 3H). ^13^C NMR (100.63 MHz, DMSO-*d_6_*) δ(ppm): 155.33; 151.38; 142.72; 137.56; 129.86; 128.46; 128.18; 126.38; 124.23; 122.52; 115.39; 99.51; 67.65; 58.81; 44.23; 21.71; 21.31; 21.16; 20.78. FTIR-ATR υ (cm^−1^): 2969; 2875; 1598; 1474; 1383; 1313; 1266; 1216; 1171; 1107; 1032; 1001; 926; 893; 839; 809; 795; 777; 767; 738; 711; 667; 621; 560; 545; 448. Elemental analysis calcd for C_24_H_24_F_6_N_2_O_4_S: C 52.36; H 4.39; N 5.09. Found: C 53.41; H 4.98; N 5.06.

2-((2,8-*bis*(trifluoromethyl)quinolin-4-yl)(hydroxy)methyl)piperidin-1-ium 2-(4-(2-hydroxyethyl)- -piperazin-1-yl)ethanesulfonate, **[MFLH][HEPES]**

Following the general procedure, sodium HEPES (0.198 g; 0.76 mmol) was added to a solution of mefloquine hydrochloride. White solid (85%). T_m_ = 134.2 °C with decomposition. ^1^H NMR (400.13 MHz, DMSO-*d_6_*) δ(ppm): 8.74 (d, J = 8.4 Hz, 1H); 8.39 (d, J = 7.2 Hz, 1H); 8.09 (s, 1H); 7.98 (t, J = 8.0 Hz, 1H); 6.54 (s, OH, 1H); 5.71 (s, 1H); 4.41 (s, OH); 3.47 (t, J = 6.4 Hz, 3H); 3.21 (dd, J^1^ = 43.2 Hz, J = 11.6 Hz, 4H); 2.81 (t, J = 12.4 Hz, 1H); 2.59 (s, 3H); 2.46-2.36 (m, 7H); 1.68-1.17 (m, 7H). ^13^C NMR (100.63 MHz, DMSO-*d_6_*) δ(ppm): 152.07; 146.48; 142.76; 129.87; 129.16; 128.12; 127.25; 126.95; 126.70; 119.87; 115.57; 69.11; 60.10; 59.77; 58.32; 54.11; 52.95; 52.47; 48.79; 45.11; 22.98; 22.06. FTIR-ATR υ (cm^−1^): 2931; 1603; 1429; 1309; 1212; 1177; 1133; 1106; 1038; 1006; 930; 881; 836; 787; 773; 738; 715; 667; 587; 547; 529; 446; 429. Elemental analysis calcd for C_25_H_34_F_6_N_4_O_5_S⋅H_2_O: C 48.7; H 5.56; N 9.09. Found: C 49.27; H 5.47; N 7.86.

#### 2.1.1. General Procedure for the Water Solubility Studies

Between 2 and 10 mg of the MFL salts was added to 1 mL of Milli-Q water and left to stir for 24 h at 37 °C. After filtration through a microporous (45 µm) syringe filter, an adequate dilution of the solution was performed and analyzed in a UV/Vis spectrophotometer at 284 nm. The absorbance value correlated with the concentration of MFL in water through the following calibration curve.

#### 2.1.2. General Procedure for the Permeability (P), Diffusion (D), and Partition Coefficient (K_d_) Measurements

The permeability measurements were conducted using a glass Franz-type diffusion cell (PermeGear) with an 8 mL reactor compartment (effective mass transfer area of 1 cm^2^). The polyethersulphone (PES-U) membranes of 150 µm thickness and 0.45 µm pore size (Sartorius Stedim Biotech, Gottingen, Germany) were placed between the two compartments and secured with a stainless-steel clamp. The receptor compartment was entirely filled with water (no bubbles) while the donor compartment contained a saturated solution of the MFL salts. Aliquots of 200 µL were removed from the donor compartment at the determined time periods (10, 20, and 30 min, and 1 and 2 h) and replenished with distilled water. The amount of MFL salts was measured by absorbance at a wavelength of 284 nm using a microplate reader (Synergy HT, Bio-TEK, Winooski, VT, USA). The experiments were performed at 37 °C, and the receptor compartment was stirred at 300 rpm using a magnetic bar to eliminate the boundary layer effect.

The permeability (P) of the MFL organic salts was calculated by the equation:

$$-\ln\left(1-\frac{2C_{t}}{C_{0}}\right)=\frac{2A}{V}\times P\times t$$

where *C_t_* is the concentration in the receptor compartment at time *t*, *C*_0_ is the initial concentration in the donor compartment, *V* is the solution volume in the two compartments, and *A* is the effective area of permeation. The permeability coefficient can be calculated from the slope of the curve −(*V*/2*A*) × ln(1 −2*C_t_*/*C*_0_) versus *t*.

The diffusion coefficient (*D*) (cm^2^.s^−1^) of solutes across the membrane was calculated according to Fick’s law of diffusion:

$$D=\frac{V_{1}V_{2}}{V_{1}+V_{2}}\times \frac{h}{A}\times \frac{1}{t}ln\left(\frac{C_{f-}C_{i}}{C_{f}-C_{t}}\right)$$

where $C_{i}$ and $C_{f}$ are the initial and final concentrations, respectively; $C_{t}$ is the concentration at time *t* of the solute in the receptor side. *V*_1_ and *V*_2_ correspond to the volume of the liquid in the donor compartment and in the receptor compartment (cm^3^), respectively. h is the thickness of the membrane (cm) and A is the effective diffusion area of the membrane (cm^2^).

The partition coefficient (*K_d_*) is defined as a measure of the solubility of the solute in the membrane and is calculated by the equation:

$$K_{d}=\frac{P\times h}{D}$$

where *P* is the permeability, h is the thickness of the membrane, and *D* is the diffusion coefficient.

#### 2.1.3. General Procedure for the Antimycobacterial Activity Studies

Briefly, 200 μL of sterile deionized water was added in all outer-perimeter wells of sterile 96-well plates (falcon, 3072: Becton Dickinson, Lincoln Park, Chicago, NJ, USA) to minimize evaporation of the medium in the test wells during incubation. The 96 plates received 100 μL of the Middlebrook 7H9 broth containing the mycobacterial cells (Difco laboratories, Detroit, MI, USA). The tested compounds were dissolved in DMSO (Sigma-Aldrich) and a serial dilution of the MFL salts was made directly on the plate. The final drug concentrations tested were 3.12–100 μg/mL. Plates were covered and sealed with parafilm and incubated at 37 °C for five days. After this time, 25 μL of a freshly prepared 1:1 mixture of Alamar Blue (Accumed International, Westlake, OH, USA) reagent and 10% Tween 80 was added in the plate and incubated for 24 h. A blue color in the well was interpreted as no bacterial growth, and a pink color was scored as growth. The MIC (minimal inhibition concentration) was defined as the lowest drug concentration, which prevented a color change from blue to pink.

#### 2.1.4. General Procedure for the Cytotoxicity Assays

Murine macrophage Raw 264.7 cells were cultured in Dulbecco’s modified Eagle’s medium (ThermoFisher Scientific, São Paulo, Brazil) supplemented with 10% (*v*/*v*) heat-inactivated fetal bovine serum, 100 IU/mL of penicillin, and 100 µg/mL of streptomycin in flat-bottomed 96-well microtiter plates (Techno Plastic Products, São Paulo, Brazil) for 24 h in 5% CO_2_ at 37 °C. At confluence, nonadherent cells were removed by washing with sterile 0.15 M phosphate-buffered saline, pH 7.2. The medium containing the compounds was added to each well, and the plate was incubated for 24 h as above. Tests containing medium alone and medium plus 1% DMSO served as controls. Cell viability was determined by the dimethylthiazol dimethyl tetrazolium bromide (MTT, Sigma-Aldrich, Burlington, MA, USA) reduction assay as recommended by the manufacturer. The assays were carried out in triplicate and performed on two separate occasions.

## 3. Results and Discussion

### 3.1. Synthesis of the MFL Salts

MFL was combined with several low-toxicity sulfonate and sulfiminium anions in order to provide a wide range of physical and chemical properties to the pharmaceutical salts. For example, dioctyl sulfosuccinate (AOT) has previously shown to display significant activity against methicillin-resistant *Staphylococcus aureus* when combined with organic cations such as ethyl glycine [56]. Moreover, saccharinate (Sac) and HEPES have yielded salts with improved solubility in water [56], while mesylate (MsO) and the (1*R*)- and (1*S*)-camsylates (CSA) have been extensively used in pharmaceutical drug formulations [57]. In fact, camphor and several derivatives are known for their bioactive properties, particularly against the *M. tuberculosis* [58,59]. Finally, tosylate (TsO) can correlate with MsO on the role of the carbon atom chain length and intramolecular interactions in the physicochemical and pharmacological properties of the MFL salts.

**Scheme 1:**
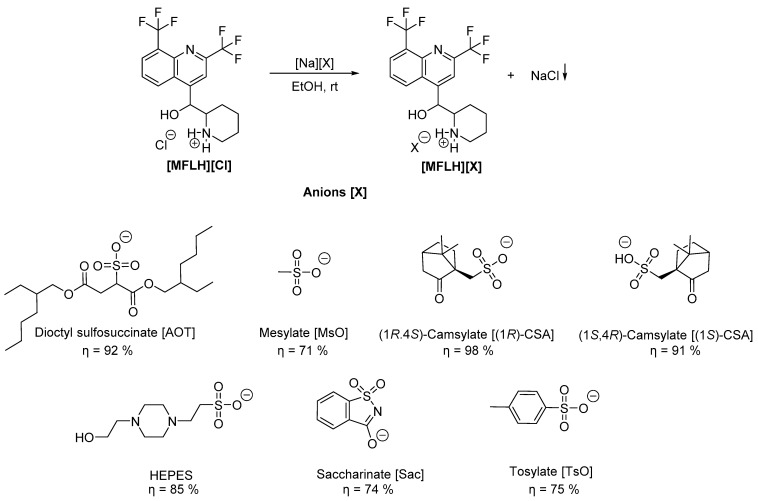
Synthetic methodology for the preparation of the MFL cationic salts by metathesis between MFL and the selected sodium sulfonates.

The MFL salts were obtained through a metathesis reaction (Scheme 1), a straightforward and well-described method for the preparation of this type of compound [60,61].

Briefly, mefloquine hydrochloride [MFLH][Cl] reacted with the respective sodium salt of the selected anion in ethanol as a solvent, at room temperature, during 16 h. Subsequently, the reaction media was filtered to remove the produced sodium chloride and the desired products were obtained in good to excellent yields and high purity after concentration under reduced pressure.

The obtained MFL salts were characterized by ^1^H and ^13^C NMR, FTIR-ATR, and elemental analysis. A strict 1.0:1.0 proportion was observed in the ^1^H NMR spectra of all MFL salts, in consonance with the desired stoichiometry, and the elemental analysis was in agreement with the theoretical values for the expected products (see ESI). The spectroscopic data, particularly NMR and FTIR-ATR spectra, were important to conclude the desired chemical structure of each MFL salt and the protonation of the MFL scaffold. In order to check the efficacy of protonation, it is possible to observe a chemical shift in the signals from the MFL cation compared to the starting compound as well as the presence of sulfonate anions in the FTIR-ATR signals compared to original sulfonic acids.

The thermal properties were determined through DSC experiments (see Figures S22–S27). The obtained melting and glass transition temperatures are gathered in Table 1, alongside the physical state of the salts at room temperature.

**Figure 1:**
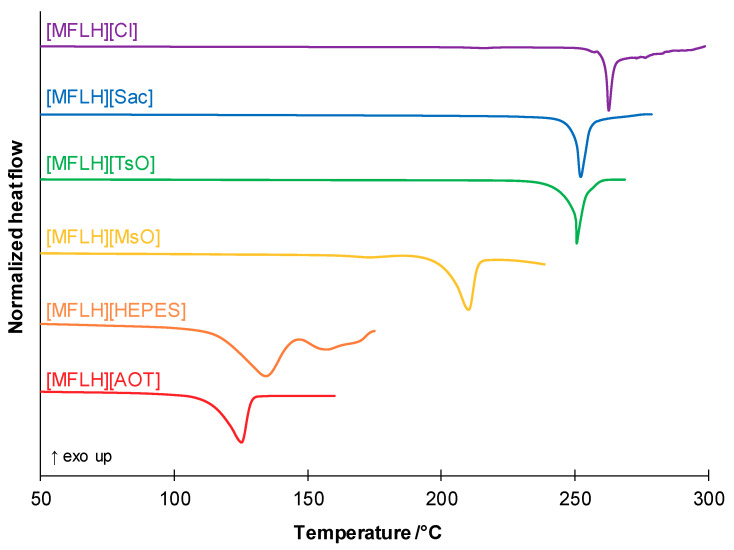
DSC first heating cycle of the tested MFL salts showing the salts’ myriad of melting temperatures in comparison with [MFLH][Cl].

All tested salts presented lower melting temperatures than [MFLH][Cl], as expected (see also Figure 1). The latter, as well as [MFLH][HEPES], appeared to decompose upon melting. Moreover, [MFLH][MsO] was the only one to retain two distinct crystalline forms, analogously to [MFLH][Cl], given by the two endothermic signals observed in the first heating cycle. However, upon first melt, all MFL salts became supercooled amorphous compounds, as no crystallization peaks were observable in the following cooling cycle. Moreover, all salts with the exception of [MFLH][HEPES] (probably due to the aforementioned decomposition) presented one glass transition temperature in the subsequent heating cycles.

### 3.2. Water Solubility and Permeability Studies

In vitro bioavailability studies were performed for all synthesized MFL salts. Typically, the formation of organic salts from pharmaceutical drugs leads to a modulation of the water solubility and partition coefficients between hydrophobic and hydrophilic media, according to the established cation–anion interactions. In the search for new lead compounds or formulations, an improved water solubility or partition through biological membranes can lead to a higher therapeutic efficiency and decreased side-effects. In fact, it is known that MFL possesses poor solubility in water, a long elimination half-life, and variations in the oral bioavailability. This substance is classified in the Biopharmaceutics Classification System (BCS) as a class II or IV drug because, associated with its low solubility, there is a lack of data related to its permeability [62,63,64]. Several strategies have been described in the literature aiming to improve MFL solubility, including the formulation of Pheroid vesicles containing MFL [65], liposome encapsulation [66], cocrystallization with different cocrystal formers [67], and the development of formulations using oil-in-water emulsions [68]. Table 2 resumes the obtained water solubility, diffusion, permeability, and corresponding partition coefficient data at 37 °C.

Almost all MFL salts presented improved in vitro bioavailability in comparison with the original [MFLH][Cl]. In terms of solubility in water (24 h shake-flask method), only [MFLH][MsO] showed improvement, probably due to the small size and solvability of the anion. Unfortunately, we were unable to grow suitable single crystals for X-ray diffraction studies, which could provide valuable insights into the crystal packing and water solvation. In addition to this enhancement in water solubility, [MFLH][MsO] also showed a slight increase in the partition coefficient (0.19), spawned from improved diffusion (0.62 cm^2^/s) and permeability (0.79 cm/s) through the synthetic membrane.

Although all remaining salts presented diminished water solubility, their diffusion and permeability parameters were improved in comparison with the original drug. The highest partition coefficient was obtained for [MFLH][TsO] (0.52), which is the least soluble studied MFL salt. These data correlate well with its excellent permeability across the membrane (4.23 cm/s) and good diffusion (1.21 cm^2^/s), which lead to the highest partition coefficient obtained.

Moreover, the two salts based on the combination of MFL with the isomers of camsylate showed very distinct results. Most probably related with very distinct interactions between the cation, the anions, and the membrane, [MFLH][(1*S*)-CSA] showed the mildest improvement in diffusion (0.22 cm^2^/s) and permeability (0.36 cm/s) values, while [MFLH][(1*R*)-CSA] presented the highest ones (1.67 cm^2^/s and 1.95 cm/s, respectively). These values mean that the former permeates the membrane at a slower rate than the latter. Nonetheless, [MFLH][(1*S*)-CSA] (0.25) has a higher K_d_ value than the [MFLH][(1*R*)-CSA] (0.18), which correlates with a slightly higher solubility in the membrane phase of the former.

The salts containing these two anions, but also [Sac] and [HEPES], are probably constituted through strong hydrogen bonds between the cation and the anion. More precisely, the oxygen atom of the carbonyl group in the structures of the camsylates and saccharinate can act as an hydrogen bond acceptor. This can also be the case with the nitrogen atoms of the piperazine ring in [HEPES]. Moreover, in this anion, the hydroxyl group can act as both hydrogen bond donor and acceptor. In this case, the partition coefficient is decreased in relation with the starting drug, despite its permeability and diffusion values being improved, ca., 10 times. Finally, the combination of MFL with [AOT] led to a highly hydrophobic salt, which precluded the execution of the partition studies.

### 3.3. Biological Activity

The antimycobacterial activity of the developed MFL salts was assessed against the susceptible *M. tuberculosis* H37RV strain (ATCC 27294) [69], using the micro-plate Alamar Blue assay (MABA) [70], a nontoxic methodology that employs a thermally stable reagent and shows good correlation with proportional and BACTEC radiometric methods [71,72]. The assays were performed in triplicate and bacteria were exposed to MFL salts in 100, 50, 25, 12.5, 6.25, and 3.12 µg/mL concentrations. The minimal inhibitory concentration (MIC) was defined as the lowest drug concentration that prevented a color change from blue to pink, and was expressed in µg/mL and µM.

The anti-mycobacterial activities and the relative decrease in inhibitory concentrations (RDIC) of the MFL salts are shown in Table 3.

With the exception of [MFLH][AOT], all salts presented the same MIC (in µg/mL). However, in molar concentrations, these salts present a slightly improved antimycobacterial activity in comparison with the starting drug. All of the starting anion sodium salts were inactive against this strain of *M. tuberculosis* (MIC > 350 µM, see Table S1), indicating that the observed biological activity originates from a synergistic effect between the MFL cation and the different organic anions.

### 3.4. Cell Viability Assay

Finally, the cytotoxicity of the MFL salts was assessed in the monocyte/macrophage-like cells Raw 264.7.

During TB infection, macrophages are the most important immune cells in the early immune response to *M. tuberculosis* once they participate in the elimination of infecting mycobacteria. However, several times, they are incapable of this and the mycobacteria colonize, survive, and grow inside macrophages [73]. Thus, due to this important role in TB pathogenesis, it is relevant to assay the cytotoxicity of anti-TB drug candidates using in vitro macrophage models. We have chosen to use Raw 264.7 cells that are monocyte/macrophage-like cells, originating from the Abelson leukemia virus-transformed cell line derived from BALB/c mice. These are the most commonly used myeloid cell line for the in vitro model for at least 40 years and they proved to be phenotypically and functionally stable until 30 passages [74].

**Figure 2:**
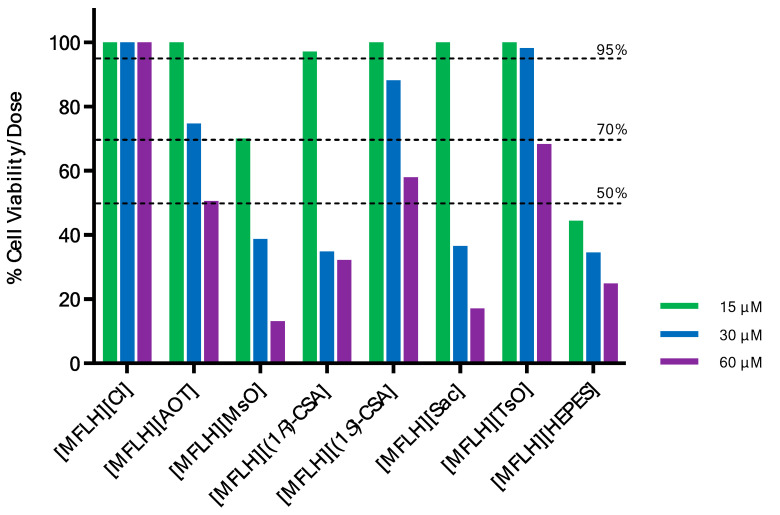
Plot of macrophages Raw 264.7 cellular viability by the MTT assay of [MFLH][Cl] and MFL salts.

The cellular viability of the MFL salts was determined by the MTT assay at three different concentrations (15 µM, 30 µM, and 60 µM) within the range of the MIC values (20.27–38.80 µM). The results are expressed as percentage of cell viability (%CV) (Figure 2 and Table S2). According to the %CV exhibited at each concentration, the compounds could be classified as noncytotoxic if the CV is as low as 95%, as moderately cytotoxic if the CV is between 70% and 94%, and as cytotoxic if the CV is lower than 50%.

At the lowest tested concentration, all salts were found to be noncytotoxic, with the exception of [MFLH][HEPES] and [MFLH][MsO]. However, in the concentration of 30 μM that is the most similar to the range of MIC values (20.27–38.80 µM), only [MFLH][Cl] and [MFLH][TsO] were noncytotoxic, whereas [MFLH][AOT] and [MFLH][(1*S*)-CSA] were moderately cytotoxic. In higher concentrations, the majority of MFL salts were cytotoxic, except for [MFLH][Cl]. It is important to highlight that [MFLH][TsO] is a promising compound because it is ca. 1.3 times more active than [MFLH][Cl] and is not cytotoxic at 15 and 30 μM.

Moreover, we also evaluated the cytotoxicity of the sodium salts used for the preparation of their corresponding MFL salts (Table S2). In general, the results indicate that these salts were not cytotoxic in all tested concentrations, except for [Na][Sac] that was cytotoxic at 60 μM. These data suggest that the association of the MFL cation with the selected organic anions tends to increase its cytotoxicity toward macrophages but also to an increase in the anti-mycobacterial activity.

## 4. Conclusions

In this work, different MFL salts based on direct protonation of the original drug by the use of organic sulfonic acids have been developed. The spectroscopic techniques such as NMR, FTIR-ATR, and elemental analysis prove the desired chemical structures. It is important to note that mefloquine mesylate ([MFLH][MsO]) presented increased water solubility in comparison with [MFLH][Cl]. Moreover, almost all salts showed improved permeability and diffusion through synthetic membranes except in the case of mefloquine docusate.

Taken together, while almost all developed MFL salts showed enhanced bioavailability in comparison with the original drug, [MFLH][(1*S*)-CSA] and [MFLH][(TsO)] seem to be the most promising salts as they display the highest activities against the susceptible *Mycobacterium tuberculosis* H37RV strain at acceptable levels of cytotoxicity toward macrophages Raw 264.7.

## Figures and Tables

**Table 1:** Physical state at room temperature and melting and glass transition temperatures of synthesized MFL salts and original [MFLH][Cl].

MFL Salts	Physical State	T_m_/°C ^a^	T_g_/°C ^b^
[MFLH][Cl]	White solid	216.3; 262.7 ^c^	- ^d^
[MFLH][AOT]	White solid	125.1	21.8
[MFLH][MsO]	White solid	173.2; 210.1	64.6
[MFLH][Sac]	White solid	252.2	102.7
[MFLH][TsO]	White solid	250.6	92.2
[MFLH][HEPES]	White solid	134.2 ^a^	- ^d^

^a^ Melting Temperature (Tm). ^b^ Glass Transition Temperature (Tg). ^c^ Decomposition Temperature. ^d^ Not observed.

**Table 2:** Solubility, diffusion (D), and permeability (P) in water, and partition coefficients (K_d_) of the MFL salts.

MFL Salts	Solubility (mg/mL)	D (×10^−6^ cm^2^/s)	P (×10^−5^ cm/s)	K_d_
[MFLH][Cl]	4.37	0.16	0.17	0.16
[MFLH][AOT]	0.03	- ^a^	- ^a^	- ^a^
[MFLH][MsO]	7.71	0.62	0.79	0.19
[MFLH][(1*R*)-CSA]	0.59	1.67	1.95	0.18
[MFLH][(1*S*)-CSA]	0.77	0.22	0.36	0.25
[MFLH][Sac]	0.21	0.60	1.18	0.30
[MFLH][TsO]	0.09	1.21	4.23	0.52
[MFLH][HEPES]	0.64	1.75	1.13	0.10

^a^ Not determined.

**Table 3:** Minimum inhibitory concentrations (MIC) and relative decrease in inhibitory concentrations (RDIC) of the MFL salts against the *M. tuberculosis*-susceptible strain H37RV.

Compounds	MIC (µg/mL)	MIC (μM)	RDIC
[MFLH][Cl]	12.5	30.1	- ^a^
[MFLH][AOT]	25	31.2	0.96
[MFLH][MsO]	12.5	26.3	1.14
[MFLH][(1*R*)-CSA]	12.5	20.5	1.47
[MFLH][(1*S*)-CSA]	12.5	20.5	1.47
[MFLH][Sac]	12.5	22.4	1.35
[MFLH][TsO]	12.5	22.7	1.32
[MFLH][HEPES]	12.5	20.3	1.48

^a^ Not determined.

## Supplementary Materials

The following supporting information can be downloaded at: https://www.mdpi.com/article/10.3390/molecules27165167/s1: Figures S1–S21: NMR (^1^H and ^13^C) and FTIR spectra of MFL salts, Figures S22–S27: DSC thermograms of MFL salts, Figure S28: Calibration curve of mefloquine in water, Figures S29–S35: Plots for diffusion, permeability, and partition coefficient studies, Table S1: Minimum inhibitory concentrations (MICs) of the anions as sodium salts, Table S2. Data of the cellular viability of macrophage Raw 264.7 cells by the MTT assay of [MFLH][Cl], MFL salts, and corresponding sodium salts.

## Sample Availability

Samples of the organic salts based on mefloquine are available from the authors.

## Abbreviations

AOT	Dioctyl sulfosuccinate
API	Active Pharmaceutical Ingredient
API–OSILs	Active Pharmaceutical Ingredient Organic Salts and Ionic Liquids
BCS	Biopharmaceutics Classification System
CSA	Camsylate
D	diffusion
DSC	Differential Scanning Calorimetry
DMSO	Dimethyl sulfoxide
FTIR	Fourier Transform Infrared
HEPES	4-(2-hydroxyethyl)-1-piperazineethanesulfonic acid
K_d_	partition coefficient
MFL	mefloquine
MFLH	mefloquine cation
MsO	mesylate
MABA	Micro-plate Alamar Blue assay
MDR	multidrug-resistant
MIC	Minimum inhibitory concentration
NMR	Nuclear Magnetic Resonance
OSILs	organic salts and ionic liquids
P	permeability
PES-U	polyethersulphone
RDIC	relative decrease in inhibitory concentrations
Sac	saccharinate
TsO	tosylate
TB	tuberculosis
T_g_	glass transition temperature
T_m_	melting temperature
US	United States
XRD	extensively drug-resistant
