# Supplementary material for: Novel Organic Salts Based on Mefloquine: Synthesis, Solubility, Permeability, and In Vitro Activity against Mycobacterium tuberculosis
Source: Molecules. 2022 Aug 13;27(16):5167. doi: 10.3390/molecules27165167 (PMC9412322; doi:10.3390/molecules27165167)
Supplement: Supplementary file 1 [file molecules-27-05167-s001.zip › molecules-1815509-supplementary.pdf]

Supporting information

Novel organic salts based on mefloquine: synthesis, solubility, permeability and *in vitro activity* against the *Mycobacterium tuberculosis*

NMR and FTIR Spectra

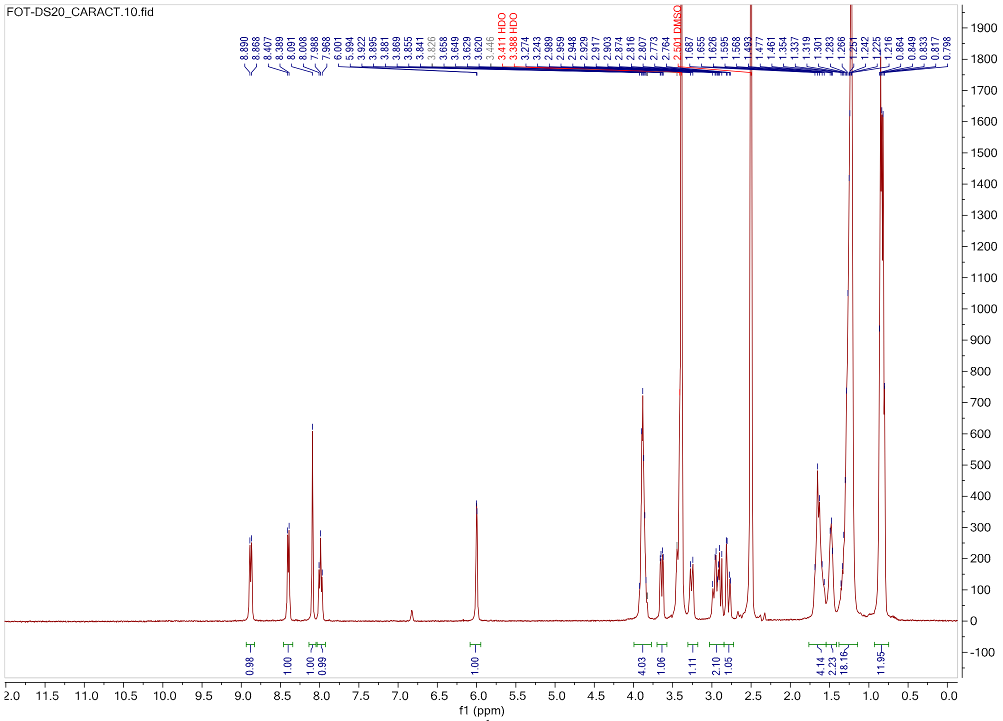

Figure S1. [MFLH][AOT] ^{1}H NMR spectrum in DMSO-d_6.

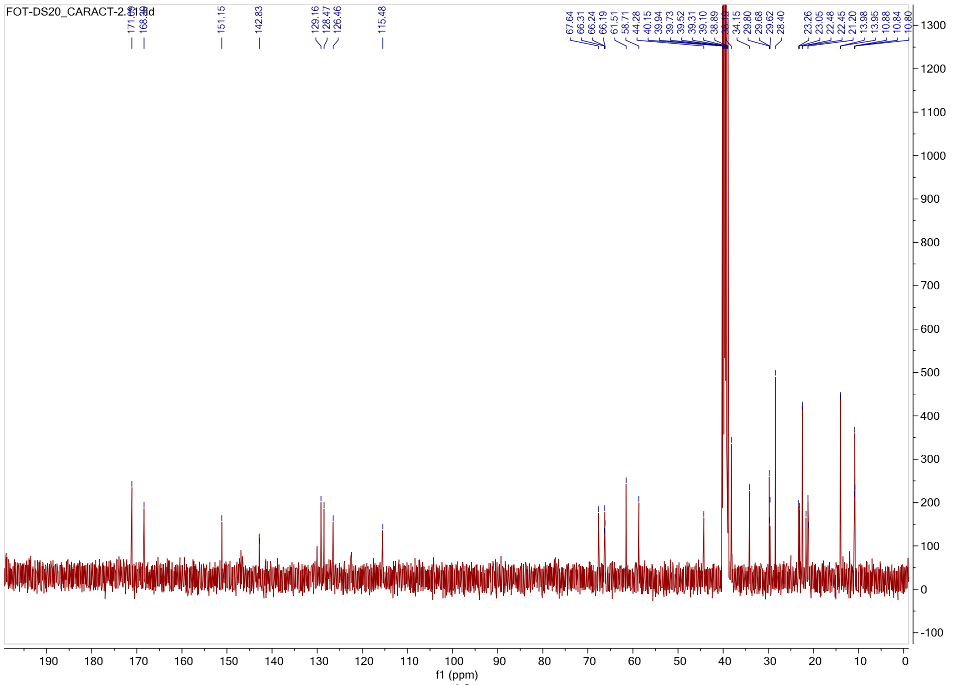

Figure S2. [MFLH][AOT] ^{13}C NMR spectrum in DMSO-d_6.

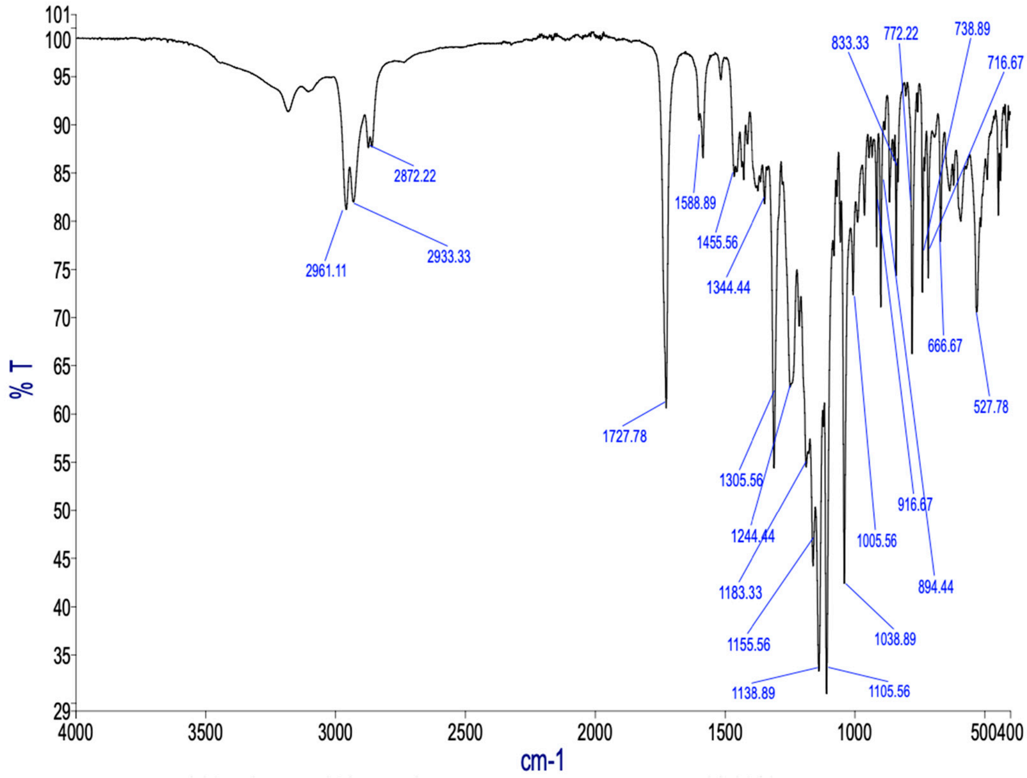

Figure S3. [MFLH][AOT] FTIR-ATR spectrum.

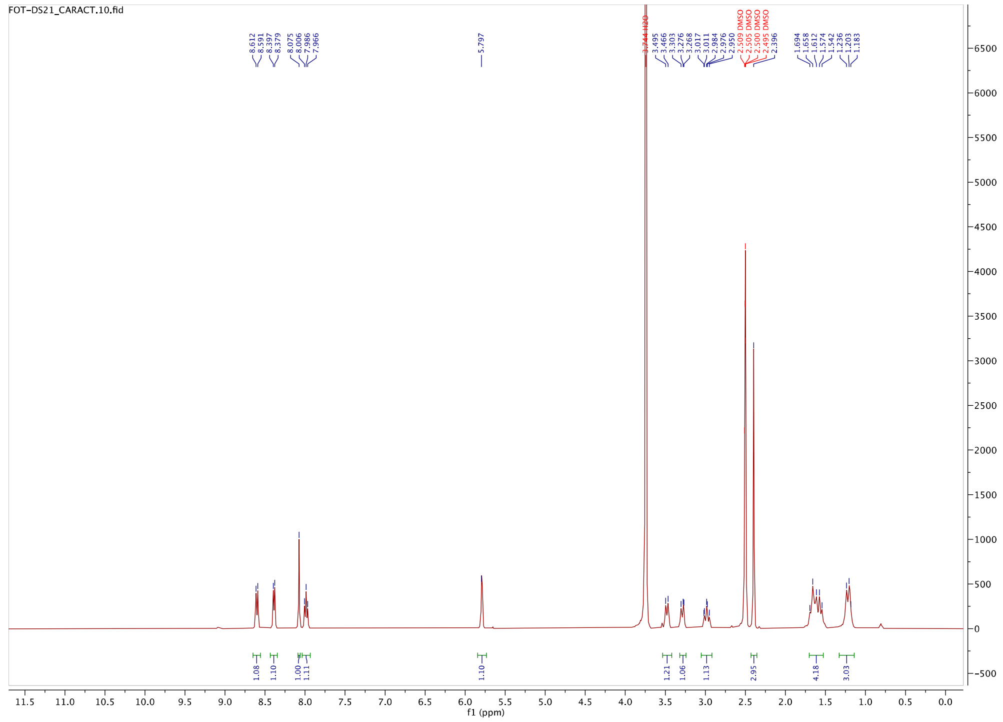

Figure S4. [MFLH][MsO] ^{1}H NMR spectrum in DMSO-d_6.

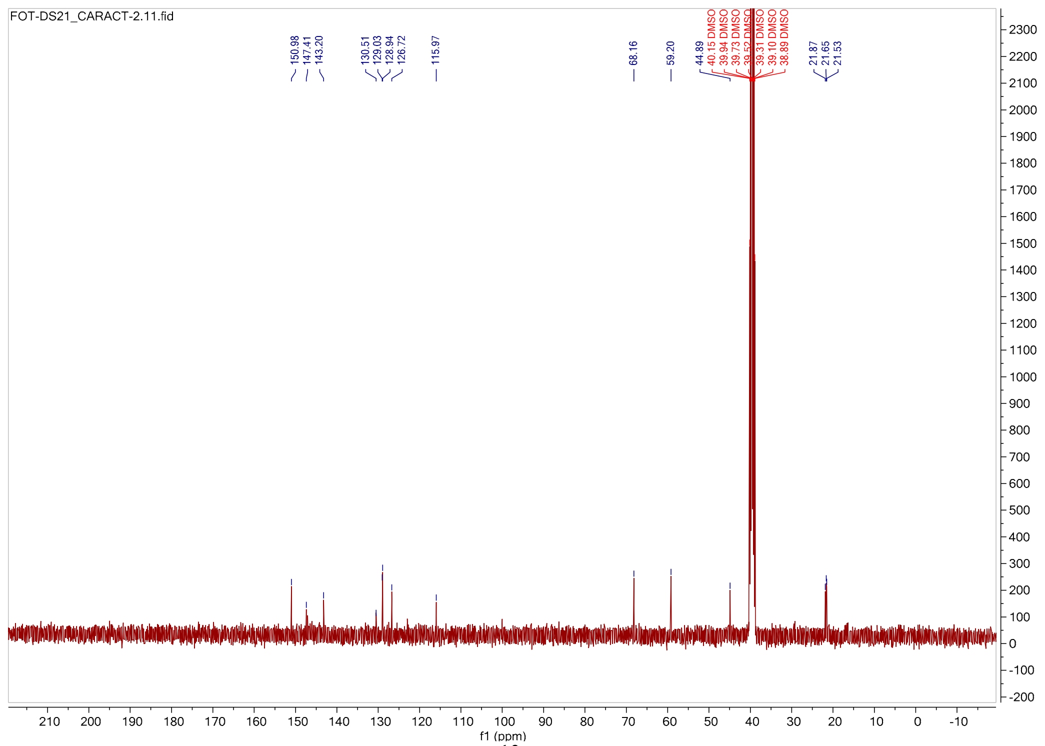

Figure S5. [MFLH][MsO] ^{13}C NMR spectrum in DMSO-d_6.

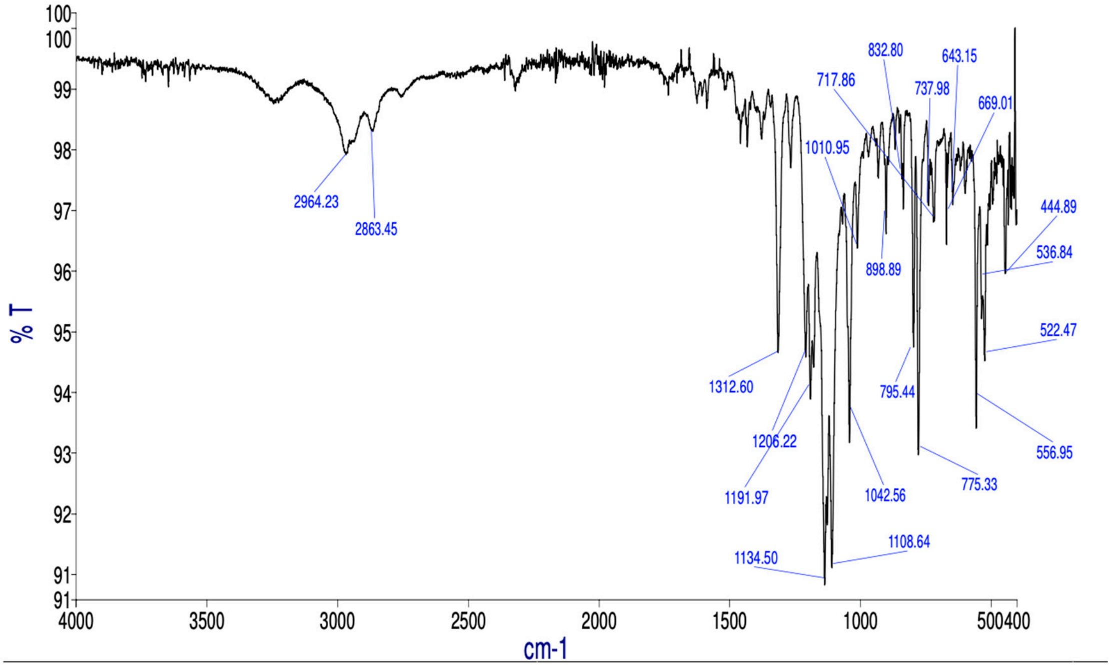

Figure S6. [MFLH][MsO] FTIR-ATR spectrum.

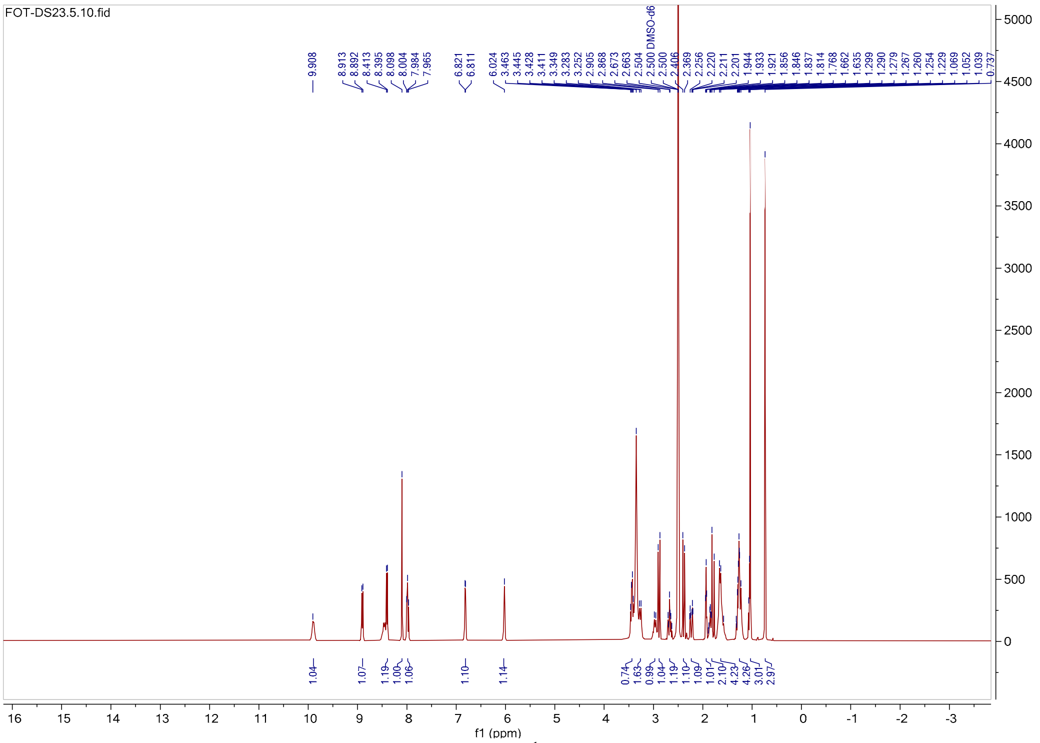

Figure S7. [MFLH][(1*S*)-CSA] ^{1}H NMR spectrum in DMSO-d_6.

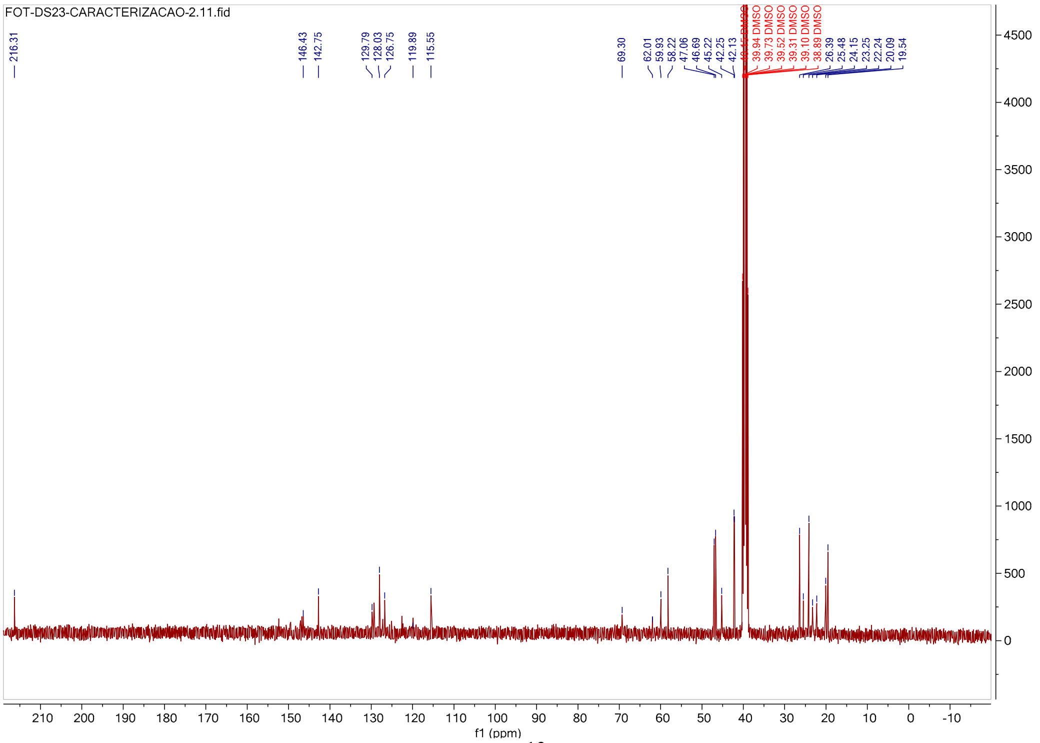

Figure S8. [MFLH][(1*S*)-CSA] ^{13}C NMR spectrum in DMSO-d_6.

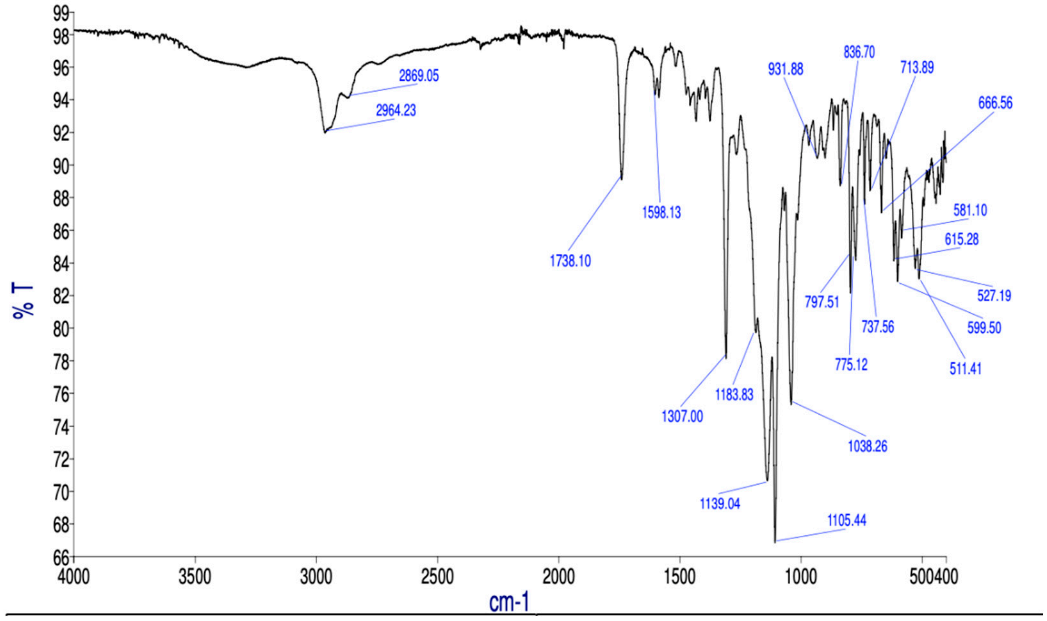

Figure S9. [MFLH][(1*S*)-CSA] FTIR-ATR spectrum.

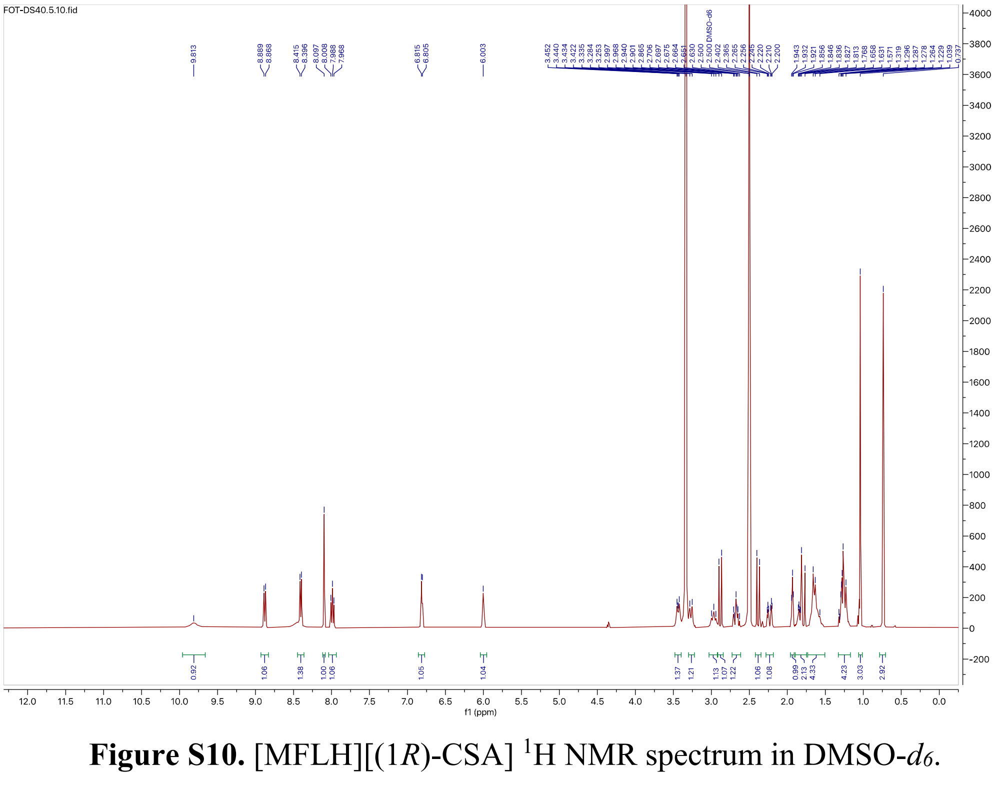

Figure S10. [MFLH][(1*R*)-CSA] ^{1}H NMR spectrum in DMSO-d_6.

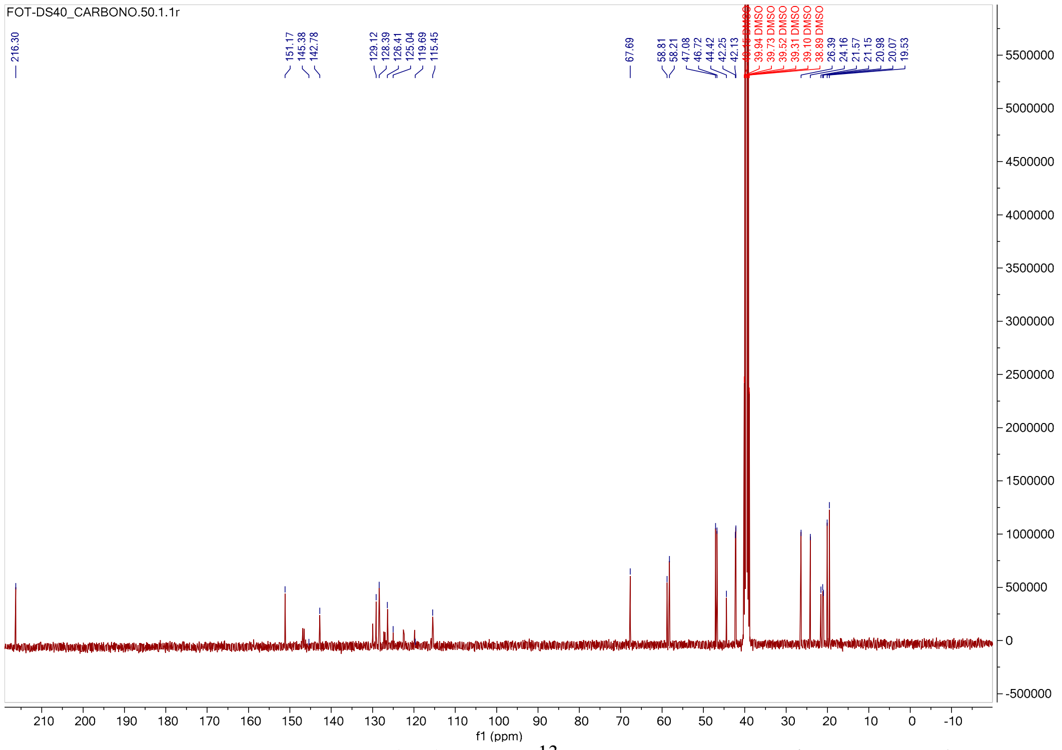

Figure S11. [MFLH][(1*R*)-CSA] ^{13}C NMR spectrum in DMSO-*d*$_6$.

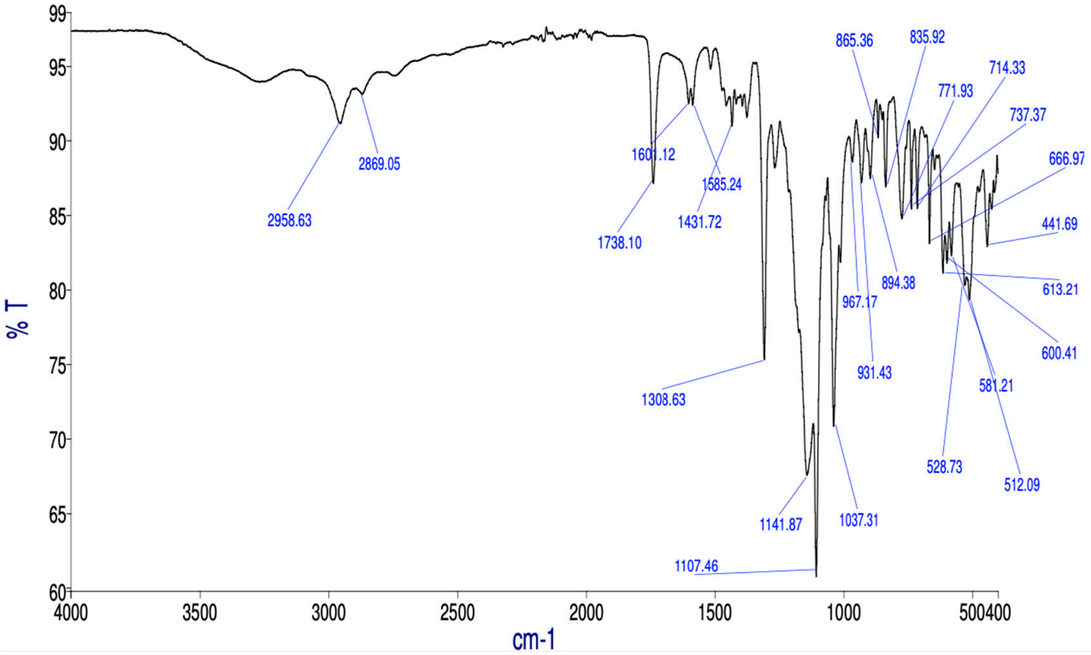

Figure S12. [MFLH][(1*R*)-CSA] FTIR-ATR spectrum.

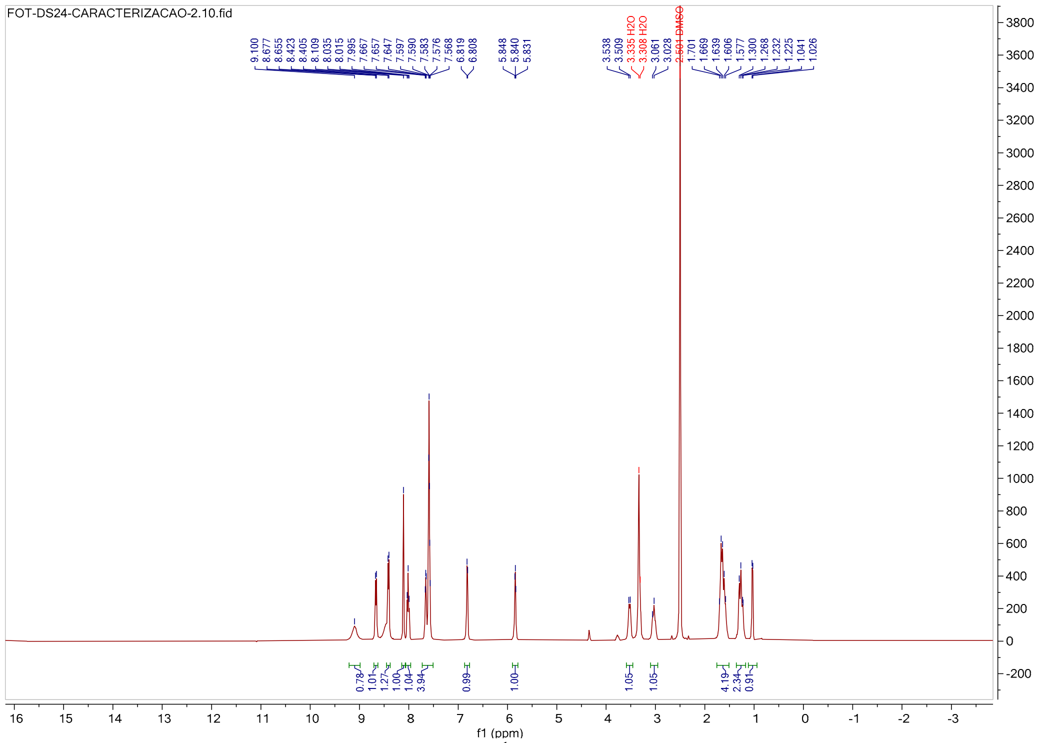

Figure S13. [MFLH][Sac] ^{1}H NMR spectrum in DMSO-d_6.

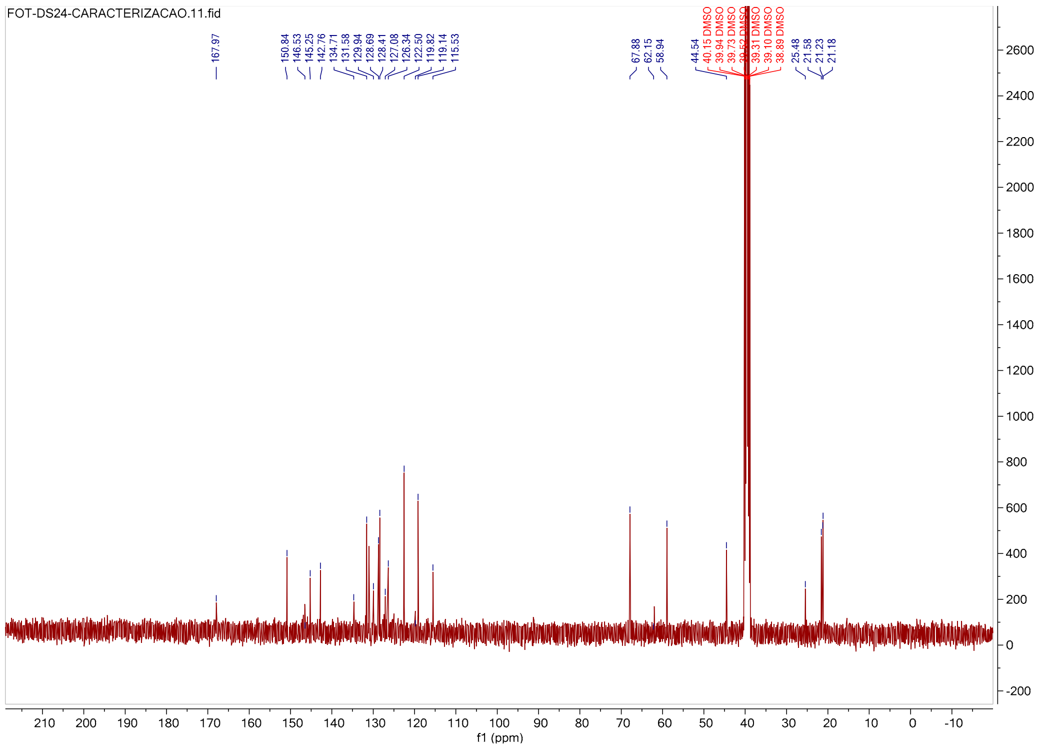

Figure S14. [MFLH][Sac] ^{13}C NMR spectrum in DMSO-d_6.

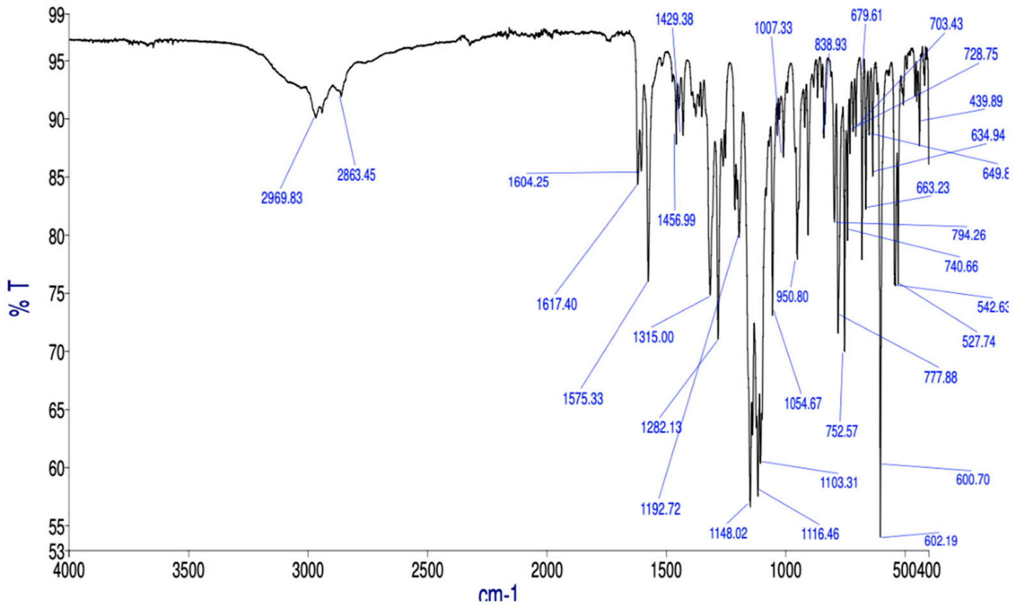

Figure S15. [MFLH][Sac] FTIR-ATR spectrum.

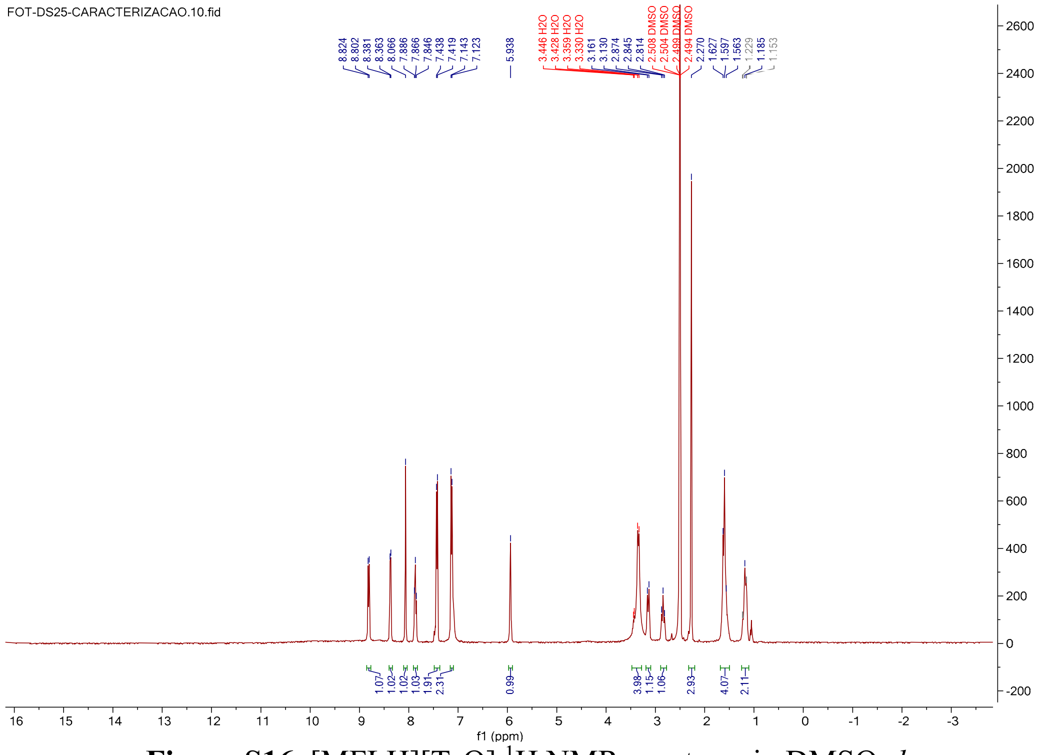

Figure S16. [MFLH][TsO] ^{1}H NMR spectrum in DMSO-d_6.

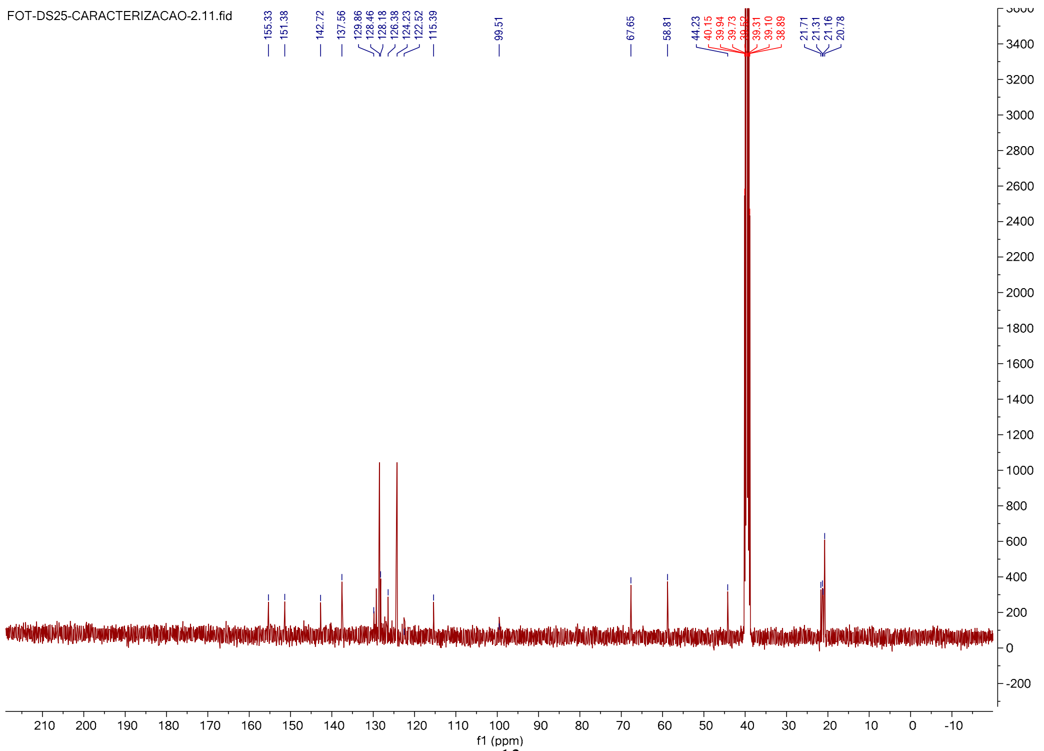

Figure S17. [MFLH][TsO] ^{13}C NMR spectrum in DMSO-d_6.

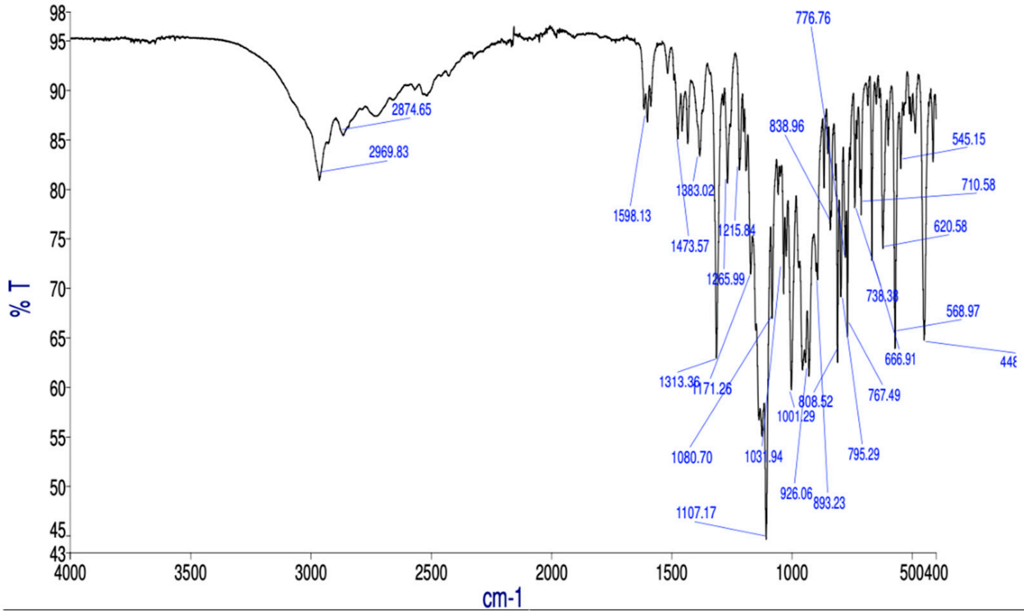

Figure S18. [MFLH][TsO] FTIR-ATR spectrum.

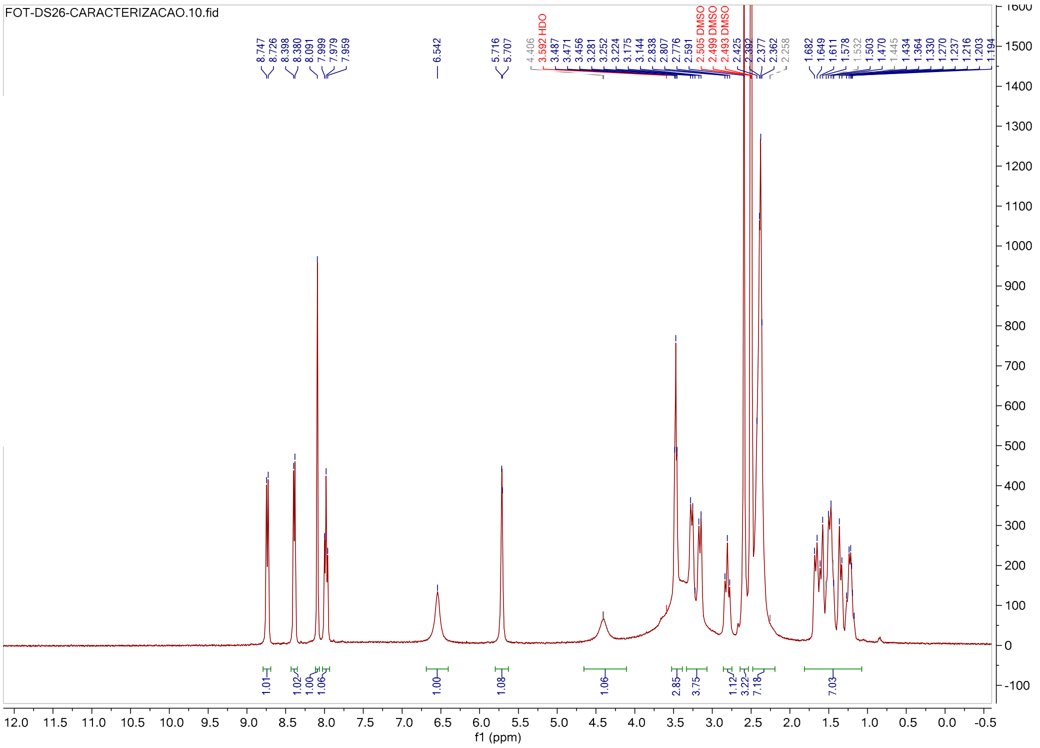

Figure S19. [MFLH][HEPES] ^{1}H NMR spectrum in DMSO-d_6.

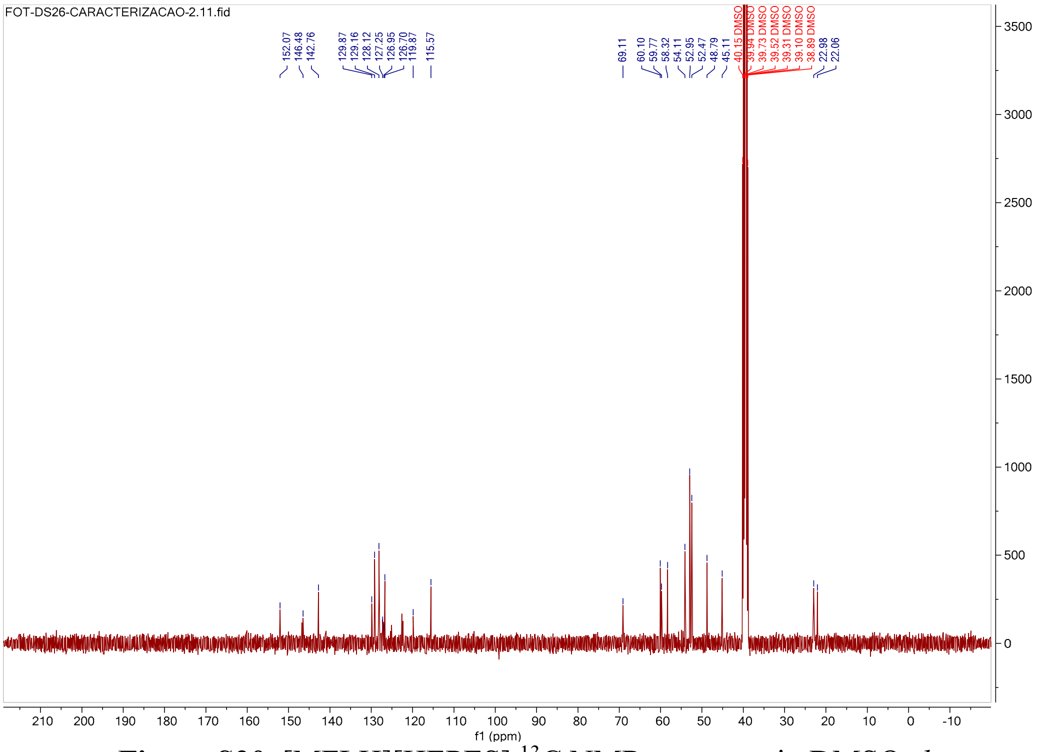

Figure S20. [MFLH][HEPES] ^{13}C NMR spectrum in DMSO-d_6.

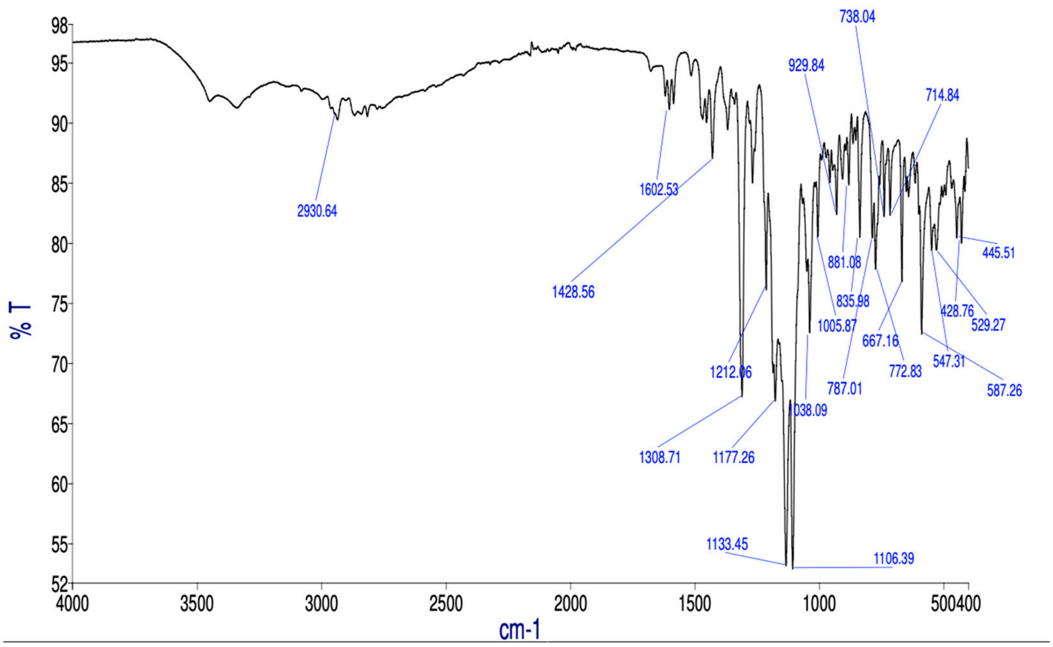

Figure S21. [MFLH][HEPES] FTIR-ATR spectrum.

DSC thermograms

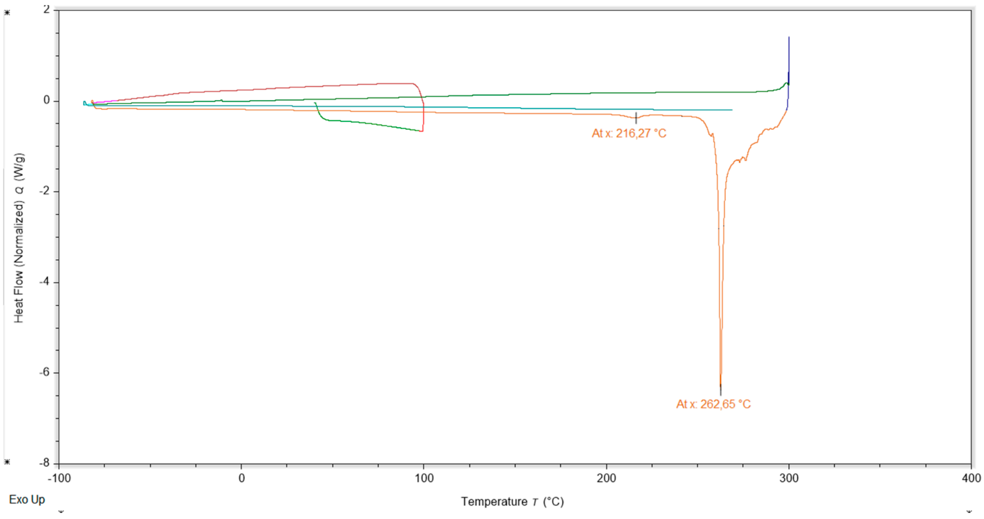

Figure S22. DSC thermogram of [MFLH][Cl].

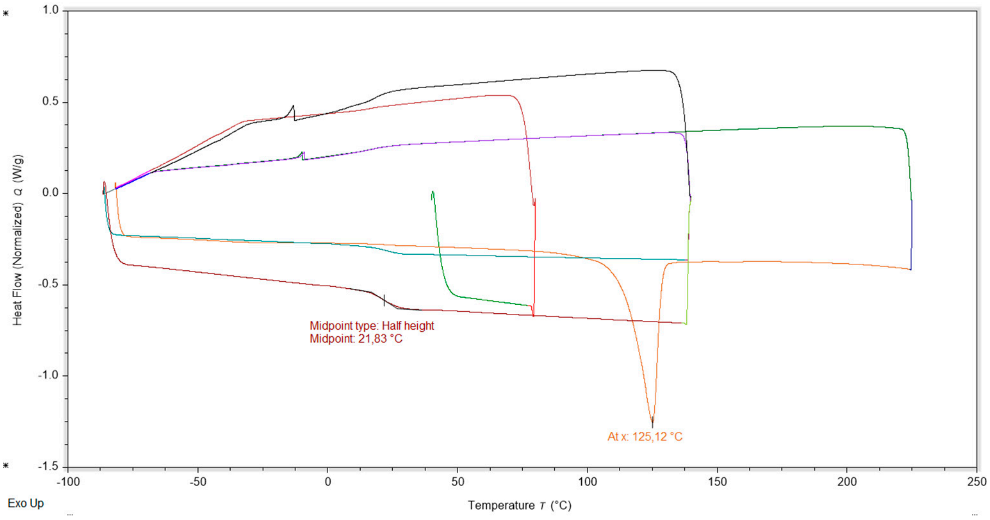

Figure S23. DSC thermogram of [MFLH][AOT].

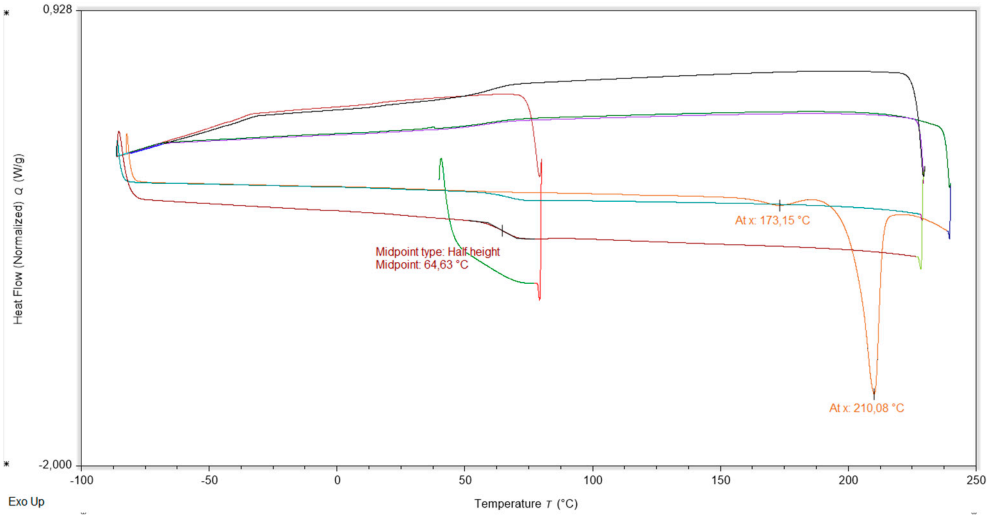

Figure S24. DSC thermogram of [MFLH][MsO].

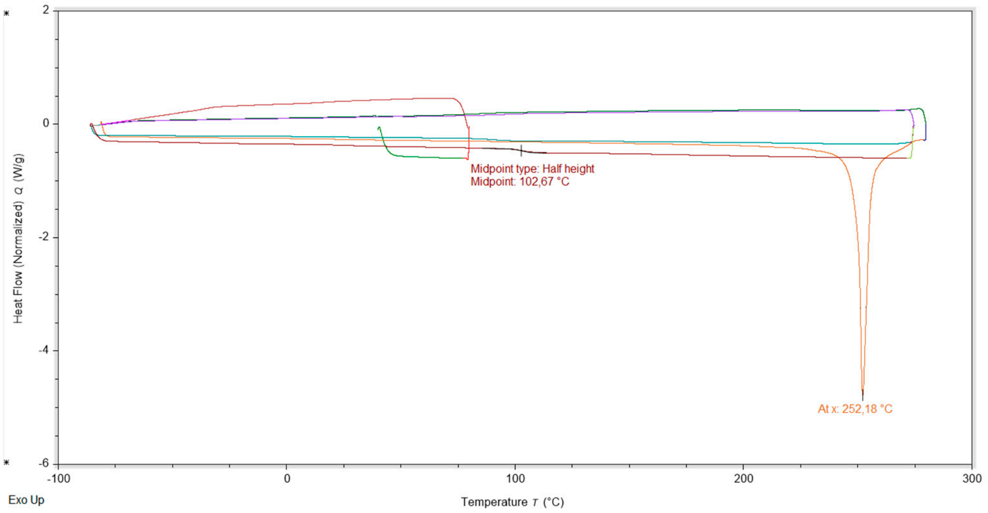

Figure S25. DSC thermogram of [MFLH][Sac].

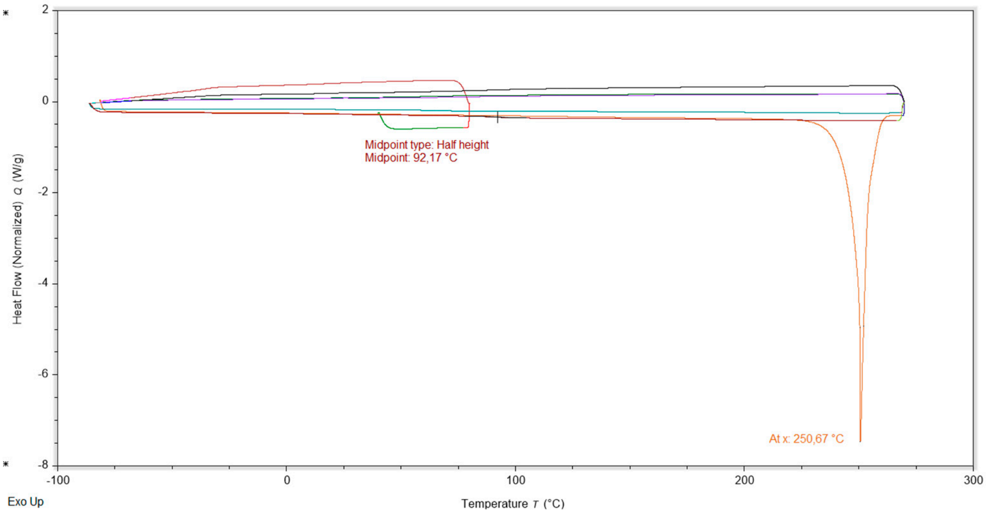

Figure S26. DSC thermogram of [MFLH][TsO].

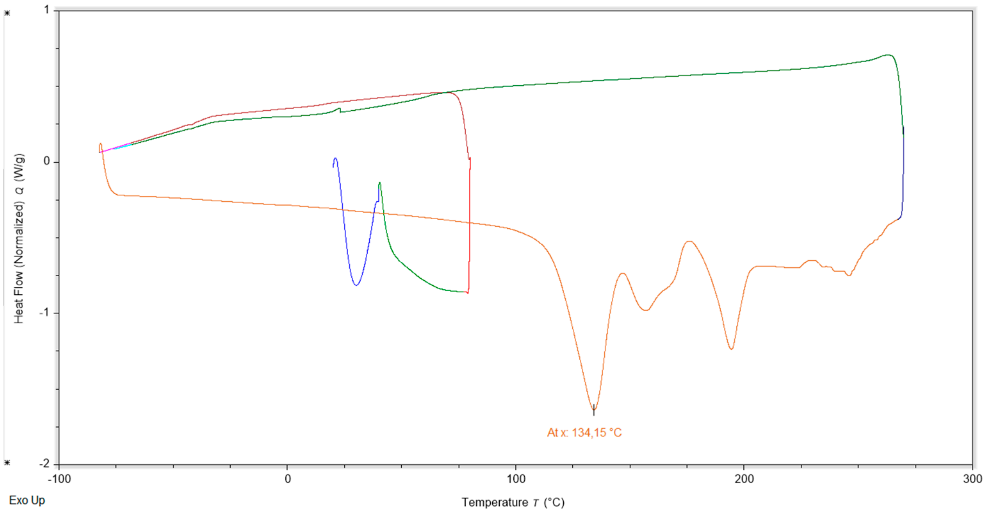

Figure S27. DSC thermogram of [MFLH][HEPES].

Water solubility studies

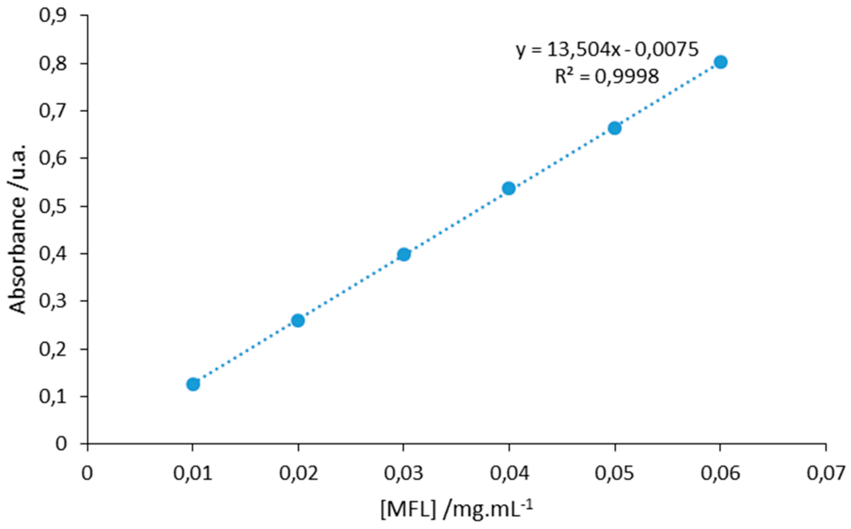

Figure S28. Calibration curve of Mefloquine in water.

Permeability (P), diffusion (D) and partition coefficient (K_d) studies

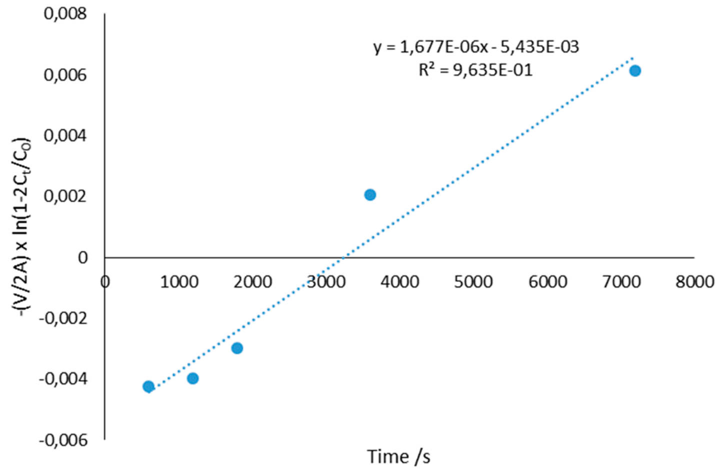

Figure S29. Plot of -(V/2A) × ln(1-2C_t/C_0) versus time (s) of [MFLH][Cl].

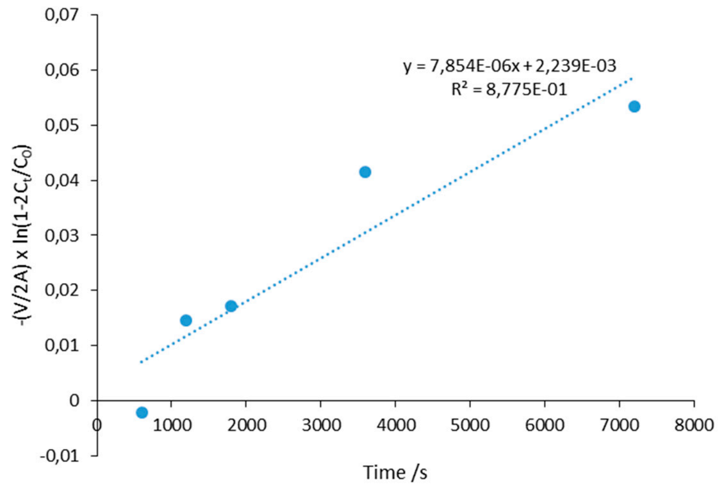

Figure S30. Plot of -(V/2A) × ln(1-2C_t/C_0) versus time (s) of [MFLH][MsO].

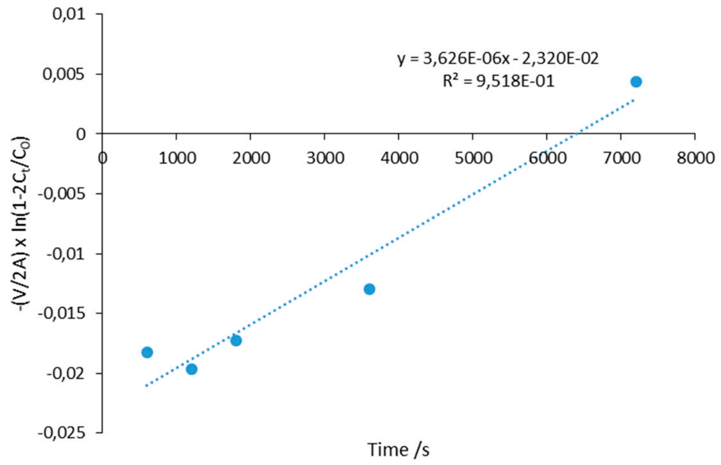

Figure S31. Plot of -(V/2A) × ln(1-2C_t/C_0) versus time (s) of [MFLH][(1*S*)-CSA].

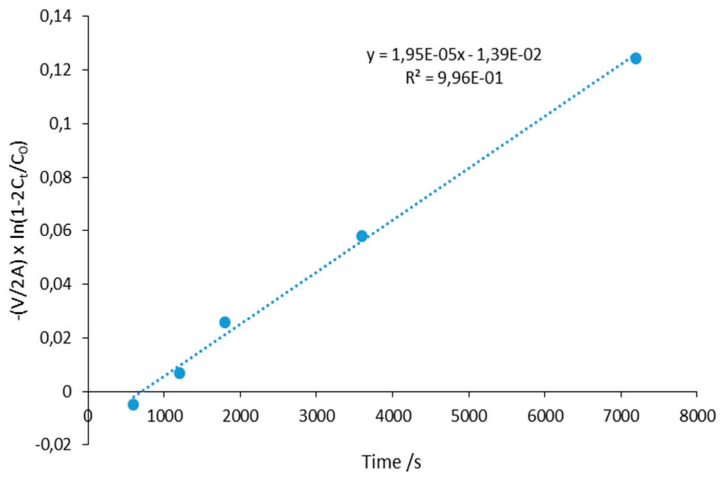

Figure S32. Plot of -(V/2A) × ln(1-2C_t/C_0) versus time (s) of [MFLH][(1*R*)-CSA].

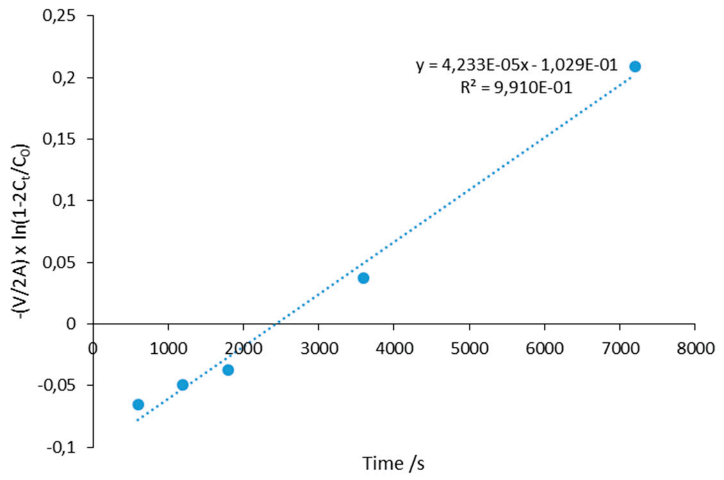

Figure S33. Plot of -(V/2A) × ln(1-2C_t/C_0) versus time (s) of [MFLH][TsO].

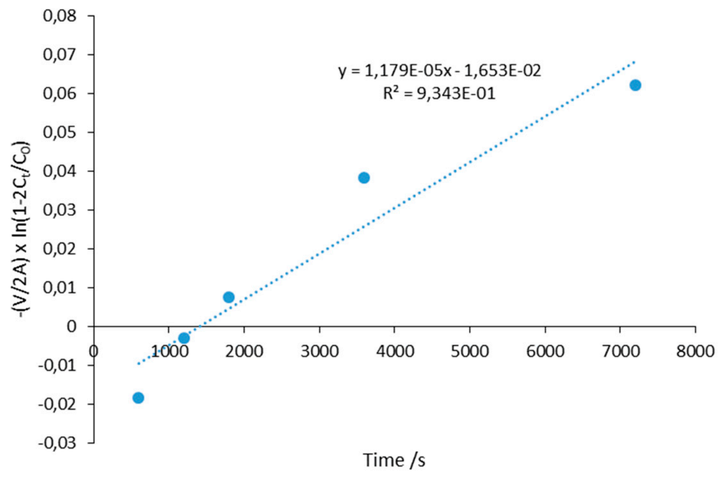

Figure S34. Plot of -(V/2A) × ln(1-2C_t/C_0) versus time (s) of [MFLH][Sac].

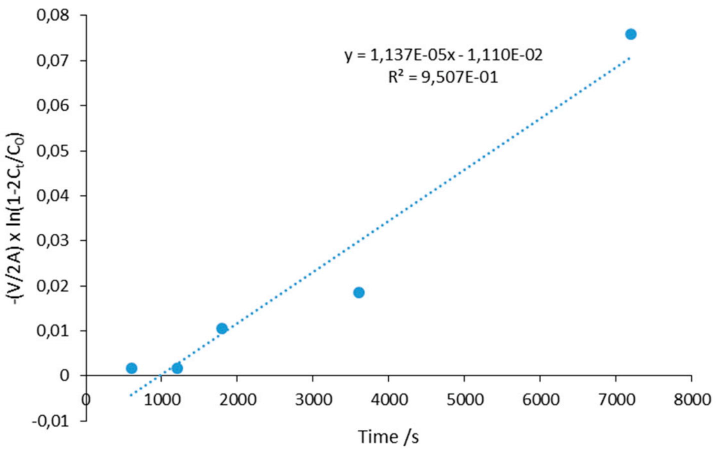

Figure S35. Plot of -(V/2A) × ln(1-2C_t/C_0) versus time (s) of [MFLH][HEPES].

Antimycobacterial activity studies

Table S1. Minimum inhibitory concentrations (MIC) of the anions as sodium salts.

**Salts**	**MIC (μM)**
Na[AOT]	> 350
Na[MsO]	> 350
Na[1*R*-CSA]	> 350
Na[1*S*-CSA]	> 350
Na[Sac]	> 350
Na[TsO]	> 350
Na[HEPES]	> 350

Cytotoxicity assays

Table S2. Data of the cellular viability of macrophage Raw 264.7 cells by the MTT assay of [MFLH][Cl], MFL salts and corresponding sodium salts.

**Compounds**	**% Cell Viability (CV)/Dose (µM)**		
	**15**	**30**	**60**
[MFLH][Cl]	100	100	100
[MFLH][AOT]	100	74.7	50.6
[MFLH][MsO]	70.0	38.7	13.2
[MFLH][(1*R*)-CSA]	97.1	34.9	32.2
[MFLH][(1*S*)-CSA]	100	88.1	58.0
[MFLH][Sac]	100	36.5	17.1
[MFLH][TsO]	100	98.2	68.3
[MFLH][HEPES]	44.4	34.5	24.9
Na[AOT]	100	100	100
Na[MsO]	100	100	100
Na[(1*R*)-CSA]	100	100	100
Na[(1*S*)-CSA]	100	100	100
Na[Sac]	100	95.9	34.8
Na[TsO]	100	100	92.5
Na[HEPES]	100	100	100
